# Neuropilin 1 mediates epicardial activation and revascularization in the regenerating zebrafish heart

**DOI:** 10.1242/dev.174482

**Published:** 2019-07-02

**Authors:** Vanessa Lowe, Laura Wisniewski, Jacob Sayers, Ian Evans, Paul Frankel, Nadia Mercader-Huber, Ian C. Zachary, Caroline Pellet-Many

**Affiliations:** 1 Centre for Cardiovascular Biology and Medicine, Division of Medicine, The Rayne Building, University College London, London WC1E 6JJ, UK; 2 Department of Developmental Biology and Regeneration, Institut für Anatomie, Universität Bern, Baltzerstrasse 2, 3012 Bern, Switzerland; 3 Department of Comparative Biomedical Sciences, Royal Veterinary College, Royal College Street, London NW1 0TU, UK

**Keywords:** Epicardium, Heart, Neuropilin, Regeneration, Zebrafish

## Abstract

Unlike adult mammals, zebrafish can regenerate their heart. A key mechanism for regeneration is the activation of the epicardium, leading to the establishment of a supporting scaffold for new cardiomyocytes, angiogenesis and cytokine secretion. Neuropilins are co-receptors that mediate signaling of kinase receptors for cytokines with crucial roles in zebrafish heart regeneration. We investigated the role of neuropilins in response to cardiac injury and heart regeneration. All four neuropilin isoforms (*nrp1a*, *nrp1b*, *nrp2a* and *nrp2b*) were upregulated by the activated epicardium and an *nrp1a*-knockout mutant showed a significant delay in heart regeneration and displayed persistent collagen deposition. The regenerating hearts of *nrp1a* mutants were less vascularized, and epicardial-derived cell migration and re-expression of the developmental gene *wt1b* was impaired. Moreover, cryoinjury-induced activation and migration of epicardial cells in heart explants were reduced in *nrp1a* mutants. These results identify a key role for Nrp1 in zebrafish heart regeneration, mediated through epicardial activation, migration and revascularization.

## Abstract (precis)

**Summary:** *nrp1a* is required for zebrafish heart regeneration in response to cryoinjury by inducing *wt1b* to promote the revascularization, and the activation and migration of the epicardium.

## INTRODUCTION

Ischemic heart disease remains the leading cause of death worldwide and, although improved therapeutic treatments have led to an increase in myocardial infarction (MI) survival rates (Cahill and Kharbanda, 2017; Roger, 2013; von Gise et al., 2011), cardiac function often remains severely compromised because adult mammalian hearts replace damaged tissue with an irreversible fibrotic scar (Dobaczewski et al., 2010; Porrello et al., 2011). This often leads to the development of chronic heart failure, further MIs and fatal arrhythmias. In contrast to mammals, zebrafish have the remarkable ability to regenerate lost or damaged cardiac tissue via cardiomyocyte proliferation and resorption of fibrotic tissue, ultimately restoring cardiac function (Chablais et al., 2011; González-Rosa et al., 2011; Jopling et al., 2010; Poss et al., 2002). Understanding the underlying mechanisms that govern zebrafish heart regeneration could identify therapeutic targets important for stimulating cardiac repair following MI in mammals.

Zebrafish heart regeneration involves a well-described, but incompletely understood, sequence of cellular processes and signaling events. The epicardium, a mesothelial cell monolayer encasing the heart, has been strongly implicated as a key regulator of the regenerative response (Cao and Poss, 2018; Masters and Riley, 2014; Zhou and Pu, 2011). Upon cardiac damage, the epicardium is activated (Schnabel et al., 2011; van Wijk et al., 2012), undergoing proliferation and secreting cytokines that stimulate cardiomyocyte cell cycle re-entry (Kikuchi et al., 2010). Autocrine and paracrine signals induce a subpopulation of epicardial cells to undergo a process known as epithelial to mesenchymal transition (EMT) (Kim et al., 2010; Lepilina et al., 2006). These epicardial cells adopt an embryonic-like gene expression profile, migrate into the injured region and differentiate into fibroblasts and mural cells that support revascularization (González-Rosa et al., 2012; Lepilina et al., 2006). Some of the signaling pathways required for the epicardial regenerative response in zebrafish have been identified and characterized. In particular, platelet-derived growth factor (PDGF)-BB and fibroblast growth factor (FGF) are both essential for epicardial EMT and coronary neovascularization in the regenerating zebrafish heart (González-Rosa et al., 2012; Kim et al., 2010; Lepilina et al., 2006). Vascular endothelial growth factor (VEGF) was also found to have a key role in the early revascularization of the injured area (Marín-Juez et al., 2016).

PDGF, FGF and VEGF are all ligands for neuropilin (NRP) transmembrane receptors (Ball et al., 2010; Pellet-Many et al., 2011; West et al., 2005). NRP1 and NRP2 share similar homology domain organization, with a large extracellular region essential for ligand binding, a single transmembrane domain and a short cytoplasmic domain (Pellet-Many et al., 2008). NRP1 was first identified as a regulator of angiogenesis and neurogenesis mediated via VEGF and semaphorin3A (Sema3aa), respectively (Gu et al., 2003; Kawasaki et al., 1999; Kitsukawa et al., 1997). In zebrafish, it is also required for vascular development and is a mediator of Vegf-dependent angiogenesis (Lee et al., 2002). Furthermore, NRPs have been shown to mediate signaling pathways for other cytokines, including PDGF, FGF and transforming growth factor (TGF)-β in various tissues in both physiological and pathological settings (Glinka and Prud'homme, 2008; Kofler and Simons, 2016; Pellet-Many et al., 2011; West et al., 2005). NRPs have also been reported to have a role in EMT in carcinomas (Adham et al., 2014; Chu et al., 2014; Grandclement et al., 2011); however, despite their known interactions with cytokines implicated in EMT, their role in the epicardial response and revascularization of the injured heart after cardiac damage is currently unknown. We used the zebrafish heart cryoinjury model (González-Rosa and Mercader, 2012) to investigate the spatiotemporal expression of the four zebrafish nrp isoforms (*nrp1a* and *nrp1b*, and *nrp2a* and *nrp2b*, orthologs of human *NRP1* and *NRP2*, respectively) in the regenerating heart. We show that all were upregulated in response to cryoinjury, with distinctive endocardial and epicardial expression during the regenerative response. NRPs are expressed in activated epicardial cells and, zebrafish expressing a truncated loss-of-function Nrp1a (*nrp1a^sa1485^*) showed impaired epicardial response to injury, indicated by the downregulation of *WT1 transcription factor b* (*wt1b*; also known as *Wilms’ tumor 1b*) expression. Epicardial explants from *nrp1a^sa1485^* fish exhibited reduced epicardial cell migration compared with wild-type fish explants. Moreover, the revascularization of the injured area was also impaired in mutant fish. We also used a rat epicardial cell line (Wada et al., 2003) to investigate potential downstream targets of NRP1 and found that downregulating the expression of NRP1 via small hairpin (sh)RNA adenovirus infection, led to a decrease in β-catenin expression, which is an important regulator of EMT (Duan et al., 2012; von Gise et al., 2011; Zamora et al., 2007). These findings reveal an essential role for Nrps in zebrafish heart regeneration, mediated by a new function for Nrp1a in epicardial activation and cell movement.

## RESULTS

### Neuropilins are upregulated during zebrafish heart regeneration

**Fig. 1.**
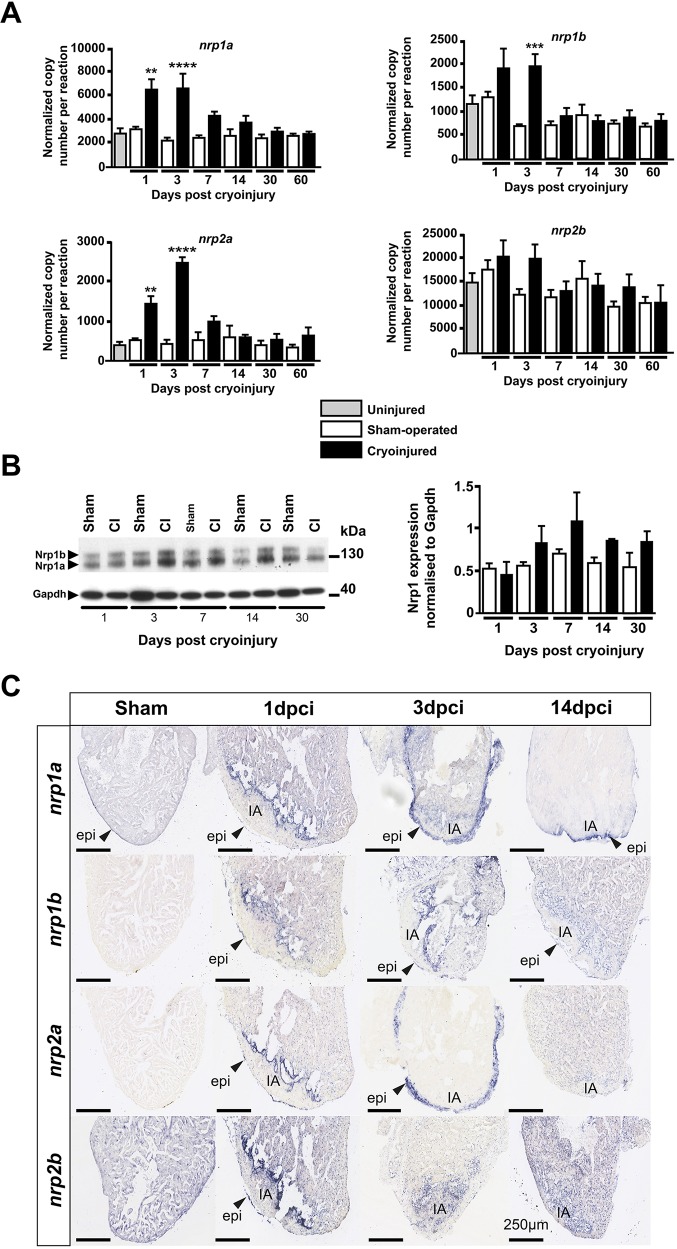
**Nrps are upregulated during zebrafish heart regeneration.** (A) Absolute qPCR analysis of nrp family genes at 1, 3, 7, 14, 30 and 60 days following cryoinjury or sham surgery. Basal expression was evaluated in uninjured hearts of age-matched wild-type fish. Bars represent normalized copy number per reaction. Data are mean±s.e.m. **P<0.01, ***P<0.005, ****P<0.001 (one-way ANOVA with Sidak's post hoc test for multiple comparisons of *n*=4 or 5 with each *n* being a pool of five ventricles). (B) Adult zebrafish ventricle lysates 1, 3, 7, 14 and 30 days following surgery, immunoblotted for Nrp1 and Gapdh (left); western blot quantification of Nrp1 protein in sham and cryoinjured ventricles 1, 3, 7, 14 and 30 days following surgery (right) (*n*=4 or 5, with each *n* being a pool of three ventricles). (C) *In situ* hybridization with digoxigenin-labeled antisense riboprobes were used to detect nrp family isoforms in sham-operated and cryoinjured adult zebrafish hearts 1, 3 and 14 dpci (*n*≥3). Arrowheads indicate gene expression within the epicardium. CI, cryoinjured; epi, epicardium; IA, injured area. Scale bars: 250 µm.

We quantified *nrp1a*, *nrp1b*, *nrp2a* and *nrp2b* mRNA levels in whole ventricles following cardiac cryoinjury by absolute RT-quantitative (q)PCR and compared their expression with that in sham-operated hearts. *Nrp1a*, *nrp1b* and *nrp2a* were upregulated 3- to 5-fold in injured hearts compared with sham-operated hearts early during the regenerative process (1 and 3 days post cryoinjury, dpci) and returned to endogenous basal levels thereafter (*P=*0.0019 and *P<*0.0001 for *nrp1a* at 1 dpci and 3 dpci, respectively; *P=*0.0007 for *nrp1b* at 3 dpci and *P=*0.0051 and *P<*0.0001 for *nrp2a* at 1 dpci and 3 dpci, respectively) (Fig. 1A). In line with a previous publication (Martyn and Schulte-Merker, 2004), *nrp2b* was the most highly expressed isoform in the heart under control conditions (Fig. 1A). However, qPCR did not show any significant *nrp2b* changes following cardiac damage, probably because any localized or cell type-specific cardiac upregulation of this isoform was masked by its high basal expression.

We also analyzed the expression of molecules implicated in Nrp-mediated signaling pathways and others with a known role in zebrafish heart regeneration (Fig. S1). Consistent with previous work, *pdgfrb*, *pdgfab* and *tgfb1a* were all upregulated early after cryoinjury (Fig. S1) (Chablais and Jazwinska, 2012; Lepilina et al., 2006). Given that NRPs are VEGF co-receptors, we examined the regulation of *vegfaa*, *vegfc* and the VEGF receptors *kinase insert domain receptor like* (*kdrl*) and *fms-related tyrosine kinase 1* (*flt1*). In accordance with previously published data (Lien et al., 2006), *vegfc* was significantly upregulated following cardiac cryoinjury (Fig. S1), but we could not detect any significant change in the expression of *vegfaa*, *kdrl* and *flt1*. In this context and at this time following the injury, *vegfc* is probably involved in inflammation and lymphangiogenesis, as previously reported (Vieira et al., 2018).

Nrp1 protein expression in zebrafish ventricles, detected by western blot, was observed as two bands of ∼130 kDa and 150 kDa, corresponding to Nrp1a [916 amino acids (aa)] and Nrp1b (959 aa), respectively (Fig. 1B). From 3 dpci and later, immunoblotting revealed an upregulation of Nrp1 proteins in the injured hearts compared with sham-operated hearts, although this did not reach statistical significance (Fig. 1B).

### Neuropilins are upregulated in the epicardium and the endocardium following cryoinjury

We used *in situ* hybridization to delineate the spatiotemporal expression of nrp family genes following cryoinjury (Fig. 1C). The specificity of the nrp RNA probes was initially analyzed in zebrafish embryos (Fig. S2), confirming expression patterns similar to previous observations in the literature (Martyn and Schulte-Merker, 2004; Yu et al., 2004). In control sham-operated adult zebrafish hearts, *nrp1a* was expressed by the epicardium and *nrp2b* was widely expressed by the myocardium (Fig. 1C). *In* *situ* hybridization revealed mRNA upregulation of all neuropilin isoforms in the epicardium and at the interface between the healthy myocardium and the injured tissue at 1 dpci. At 3 dpci, both *nrp1a* and *nrp2a* were strongly and more widely upregulated by the activated epicardium, whereas *nrp1b* was expressed at the injury border. At 14 dpci, strong expression of *nrp1a* persisted in the epicardium adjacent to the injured area, and *nrp1b* was localized in the epicardium and the endocardium contiguous to the injured area. By 60 dpci, when heart regeneration was largely complete, expression of all *nrp* isoforms had returned to basal expression levels, which correlated with the gene expression data (data not shown).

**Fig. 2.**
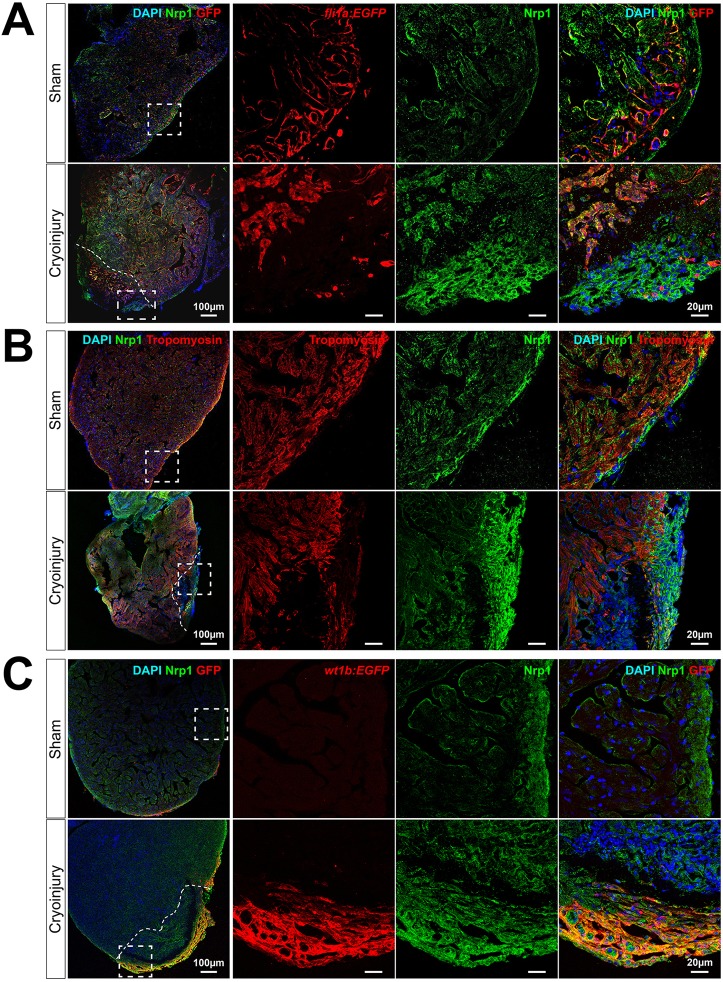
**Nrp1 is expressed by the endocardium and the epicardium in sham and cryoinjured hearts.** (A-C) Immunostaining of 7 days post sham-operated (upper rows) and cryoinjured (lower rows) hearts. *Tg(fli1a:EGFP)^y1^* (A), wild-type fish immunostained for tropomyosin (B) and *tg(wt1b:EGFP)^li1^* (C) zebrafish hearts were used to identify endothelium, myocardium and activated epicardium, respectively (*n*≥3). The merged images are displayed with DAPI nuclei staining. Dashed boxes denote the location of enlarged images (right panels); dashed lines indicate the injury interface. Scale bars: 100 µm; 20 µm in enlarged images.

To identify the Nrp1-expressing cells within the regenerating heart, we used co-immunofluorescent staining with specific endothelial, myocardial and epicardial markers. In *tg(fli1a:EGFP)^y1^* zebrafish, in which EGFP is specifically expressed in vascular endothelial cells, Nrp1 was co-expressed by *fli1a*-EGFP-expressing cells in sham hearts, consistent with expression of Nrp1 by coronary vessels and endocardium (Fig. 2A, upper row). Nrp1 expression was also evident in *fli1a*-EGFP-expressing neovasculature and activated endocardium at the injured area in cryoinjured hearts (Fig. 2A, lower row). These observations were supported by immunostaining of *tg(kdrl:mCherry)^s896^* transgenic fish, in which mCherry expression is driven by the promoter for the endothelial VEGF receptor, *kdrl*. Nrp1 immunostaining at 7 dpci in *tg(kdrl:mCherry)^s896^* fish showed co-expression of mCherry-positive endocardium and Nrp1 (Fig. S3). Furthermore, neovascularization was observed as early as 1 dpci in *tg(fli1a:EGFP)^y1^* fish, consistent with previous findings (Marín-Juez et al., 2016), and these early neovessels also exhibited Nrp1 expression (Fig. S4). Nrp1 expression by tropomyosin-positive cardiomyocytes was low in control sham-operated hearts (Fig. 2B, upper row). However, following cryoinjury, Nrp1 was expressed by a small population of cardiomyocytes located within the subepicardial layer at the lesion (Fig. 2B, lower row).

Epicardial expression of Nrp1 was examined in *tg(wt1b:EGFP)^li1^* zebrafish, in which EGFP expression is controlled by the promoter for the activated epicardial marker, *wt1b*. No detectable expression of EGFP was observed in sham-operated control hearts of *tg(wt1b:EGFP)^li1^* zebrafish (Fig. 2C, upper row). By contrast, we observed high levels of colocalization between Nrp1 and EGFP in the epicardium covering the lesion in cryoinjured *tg(wt1b:EGFP)^li1^* zebrafish (Fig. 2C, lower row). Furthermore, immunofluorescent staining of Wt1 and Nrp1 in cryoinjured wild-type zebrafish revealed strong colocalization of Wt1 with Nrp1 in the epicardium adjacent to the injured area at 3 dpci (Fig. S5).

### *Nrp1a* mutant zebrafish (*nrp1a^sa1485^*) display delayed heart regeneration following cryoinjury

**Fig. 3.**
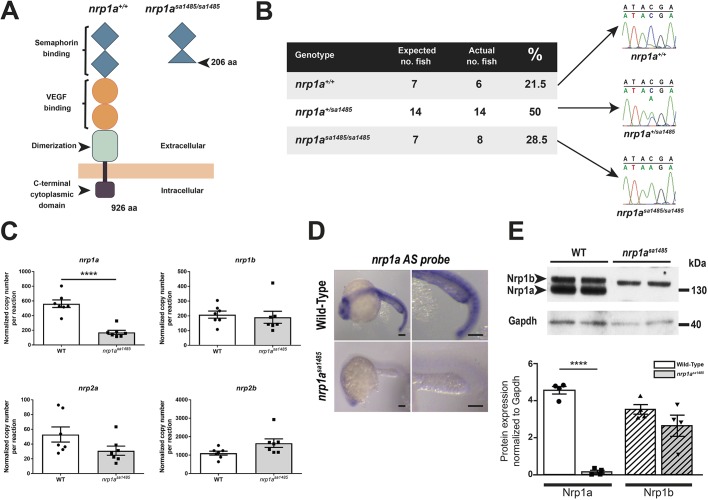
**Characterization of *nrp1a^sa1485^* mutant fish.** (A) Structure of Nrp1a in wild-type (*nrp1a^+/+^*) (left) and *nrp1a^sa1485^* mutant fish (right). The point mutation results in the generation of a premature stop codon at aa 206, resulting in a truncated Nrp1a fragment. Blue diamonds indicate CUB (a1 and a2) domains, orange circles indicate FA58C (b1 and b2) domains, the green square indicates the MAM domain and the brown square indicates the C-terminal domain. (B) Sequencing chromatograms of wild-type fish, heterozygous *nrp1a^sa1485/+^* and homozygous *nrp1a^sa1485/sa1485^* mutant fish. An early stop codon (nonsense mutation) TAA, replaces the wild-type TAC codon at aa 206. The genotypes of 14 zebrafish embryos 48 h post fertilization (hpf) were compared against the expected Mendelian ratio after heterozygous fish incross. (C) Absolute RT-qPCR of wild-type (WT; black circles, white bar) or *nrp1a^sa1485^* homozygous mutant (black squares, gray bar) uninjured adult zebrafish hearts under basal conditions. *nrp1a* expression was significantly decreased in *nrp1a^sa1485^* samples, suggesting nonsense-mediating decay. ****P<0.0001 (two-tailed *t*-test; *n*=7 with each *n* being a pool of three ventricles). Data are means of normalized copy numbers per reaction±s.e.m. (D) *Nrp1a* antisense (AS) *in situ* hybridization of wild-type (upper row) or *nrp1a^sa1485^* homozygous mutant (lower row) embryos 24 hpf. *Nrp1a* expression was clearly decreased in *nrp1a^sa1485^* samples. Scale bars: 50 μm. (E) Western blot of wild-type (WT) or *nrp1a^sa1485^* homozygous mutant uninjured adult zebrafish ventricle lysates (top). Lysates were immunoblotted with an antibody targeting the Nrp1 cytoplasmic domain and Gapdh. Note the absence of C terminus detection of Nrp1a in the *nrp1a^sa1485^* samples. Western blot quantification of Nrp1a (plain bars, circles for wild type, squares for *nrp1a^sa1485^*) and Nrp1b (striated bars, upward triangles for wild type, downward triangles for *nrp1a^sa1485^*) normalized to Gapdh (one-way ANOVA with Sidak's post hoc test for multiple comparisons of *n*=4), confirming the significant reduction in Nrp1a expression (****P<0.0001; plain white bar for wild type versus plain gray bar for *nrp1a^sa1485^*), whereas Nrp1b was not significantly different between wild type (white striated bar) and *nrp1a^sa1485^* (gray striated bar) (*P=*0.219) (bottom).

**Fig. 4.**
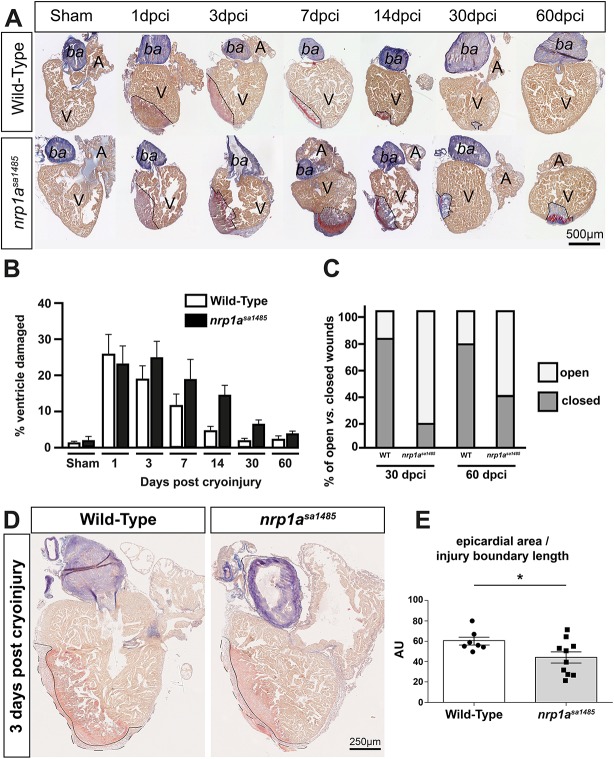
**Cardiac regeneration is delayed in *nrp1a^sa1485^* mutants following cryoinjury.** (A) Heart sections from wild-type (top) and *nrp1a^sa1485^* mutant (bottom) fish obtained at 1, 3, 7, 14, 30 and 60 dpci and stained with AFOG to identify the injured region. Dashed lines indicate the interface between cryoinjured and healthy tissue. (B) Cryoinjured areas were measured and represented as mean percentage of total ventricle area±s.e.m. *P*<0.05 (two-way ANOVA with Sidak's post hoc test for multiple comparisons of *n*=4-8). (C) A closed wound is one in which compact myocardium recovers following cryoinjury and encases the scar tissue; by contrast, a scar exposed to the surface is defined as an open wound. Wound closure was examined in wild-type (WT) and *nrp1a^sa1485^* mutant hearts at 30 and 60 dpci and open versus closed wounds were expressed as a percentage of the total number of hearts (*n*=4-8). (D,E) AFOG staining of wild-type and *nrp1a^sa1485^* mutant hearts at 3 dpci (D) used to evaluate epicardial thickness and injury boundaries to calculate the epicardial area normalized to the length of the injury boundary (continuous line), quantified in E. Dashed line represents outer boundary of epicardial area. *P<0.05 (two-tailed *t*-test of *n=*7 for wild-type and *n*=10 for *nrp1a^sa1485^* hearts). A, atrium; ba, bulbus arteriosus; V, ventricle.

The marked upregulation of *nrp1* mRNA and protein at the borders of healthy and cryoinjured myocardium, and the expression of Nrps by the endocardium and the activated epicardium suggested a role for Nrps in heart regeneration, particularly in the activated epicardium. Given the striking epicardial and endocardial expression of *nrp1a* after myocardial injury, we assessed the role of this isoform using the *nrp1a^sa1485^* homozygous mutant zebrafish. This mutant carries a nonsense mutation (tyrosine to ochre, TAA) at aa 206 (full length, 916 aa) in the second CUB domain of the *nrp1a* gene, resulting in the generation of a nonfunctional and truncated soluble N-terminal fragment (Fig. 3A). Given that the mutation occurs in the second CUB domain (also called a2), the binding of both Vegf and Semaphorin 3A are predicted to be impaired in this mutant. The binding domain of other ligands, such as Fgf, Pdgf and Tgf, have not yet been fully characterized, but it is known the deletion probably prevents receptor oligodimerization or oligomerization and any resulting downstream signaling. Thus, the loss of *nrp1a* in these mutant fish has been shown to induce axons to misproject to the dorsal and anterior dorsal zone protoglomerulus (Taku et al., 2016). *Nrp1a^sa1485^* mutant fish were viable, born at expected Mendelian ratios (Fig. 3B), displayed no obvious abnormal phenotype (Fig. S6A), and their body lengths and heart sizes were similar to those of wild-type fish (Fig. S6B-D). In the *nrp1a^sa1485^* fish, *nrp1a* endogenous basal expression was significantly reduced at both the mRNA (*P<*0.0001) (Fig. 3C,D) and protein (*P<*0.0001) (Fig. 3E,F) levels, suggesting nonsense-mediated decay, whereas the other nrp family isoforms (*nrp1b*, *nrp2a* and *nrp2b*) were not significantly altered (*nrp1b*, *P=*0.71; *nrp2a*, *P=*0.09; and *nrp2b P=*0.06) (Fig. 3C). Acid Fuchsin Orange G (AFOG) staining was used to quantify the extent of the injury in both wild-type and *nrp1a^sa1485^* fish over 60 days (Fig. 4A). Following cryoinjury, the extent of lesions in *nrp1a^sa1485^* and wild-type fish hearts was similar, affecting 22.6±5.2% (mean±s.e.m.) and 25.2±5.5% of the ventricle, respectively (Fig. 4B). By 60 dpci, the injured area was almost cleared and new healthy myocardium had replaced the damaged tissue in wild-type fish (Fig. 4A,B). A reduction in the extent of heart repair was observed from as early as 7 dpci in *nrp1a^sa1485^* hearts compared with wild-type hearts (Fig. 4A,B). Whereas fibrin deposits (red staining in injury area) were mostly cleared from the injury scars in wild-type fish by 14 dpci, fibrin deposits were still evident at 30 and 60 dpci in *nrp1a^sa1485^* mutants (Fig. 4A). Quantification of the size of the cryoinjuries revealed an overall significant delay in the regeneration of mutant hearts compared with wild-type hearts (two-way ANOVA with Sidak's post hoc test for multiple comparisons, *P*=0.0208) (Fig. 4B). Differences between *nrp1a^sa1485^* and wild-type hearts were also observed in the regeneration of the cortical layer and wound closure. In wild-type zebrafish hearts, regeneration typically led to formation of a continuous layer of cardiomyocytes enclosing the residual collagen scar, resulting in complete wound closure in the advanced stages of regeneration (30 and 60 dpci). By contrast, a larger proportion of mutant hearts at 30 and 60 dpci retained open wounds without complete closure of the lesion (Fig. 4C). We also quantified the surface area of the epicardium normalized to the length of the injury border to determine relative epicardial thickness in wild-type and *nrp1a^sa1485^* mutants (Fig. 4D,E). We found a modest but significant reduction in epicardial thickness in mutant hearts at 3 dpci (*P*=0.048), suggesting a decrease in epicardial activation.

### Revascularization of the cryoinjured heart tissue is impaired in *nrp1a^sa1485^* mutant zebrafish

**Fig. 5.**
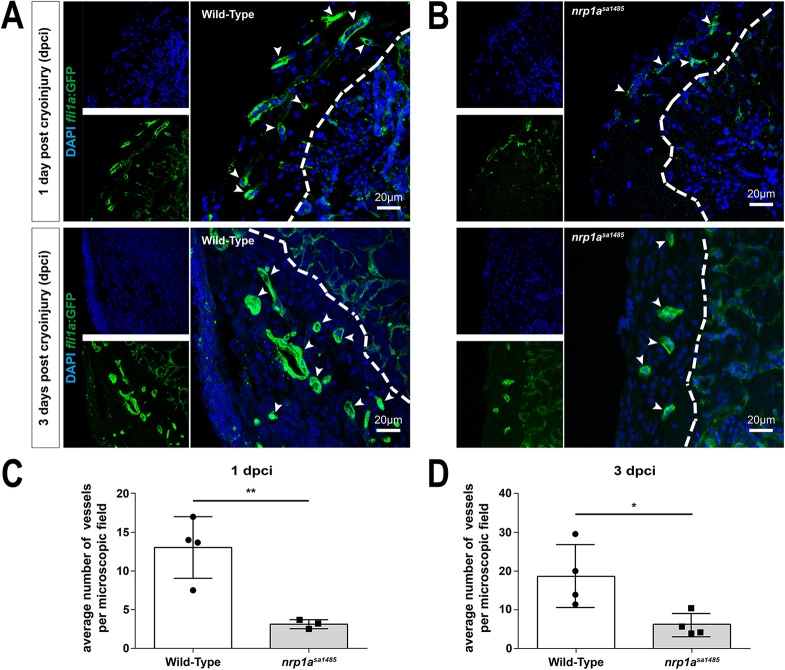
**Neovascularization of the cryoinjured** **area is impaired in *nrp1a^sa1485^* mutants.** (A,B) Blood vessels in either wild-type (A) or *nrp1a^sa1485^* (B) *tg(fli1a:EGFP)^y1^* zebrafish at 1 (upper row) and 3 (lower row) dpci were identified in heart sections using GFP immunofluorescence in vascular structures. Heart sections were also counterstained with DAPI. Smaller images (left) represent DAPI staining (blue) and GFP staining (vessels, green) only; larger images (right) are the merged images. The white dashed line delineates the border of the area of injury. White arrowheads indicate blood vessels. (C,D) GFP-positive vessels were quantified at 1 dpci (C; **P<0.01, two-tailed *t*-test of *n*=3 and 4) and 3 dpci (D; *P<0.05, two-tailed *t*-test of *n*=4) for wild-type (white bars) versus *nrp1a^sa1485^* (gray bars) hearts. Individual data points (circles for wild type and squares for *nrp1a^sa1485^*) represent individual hearts, each averaged from vessel counts in three to four different sections covering the injury site.

The impact of loss of functional Nrp1 on revascularization in cryoinjured hearts was examined by generating *nrp1a^sa1485^* mutants in *tg(fli1a:EGFP)^y1^* zebrafish, in which endothelial-specific Green Fluorescent Protein (GFP) expression is driven by the *fli1a* promoter. Angiogenesis occurs rapidly following heart injury in zebrafish, with a marked neovascular response evident as early as 1 dpci (Marín-Juez et al., 2016). Therefore, we compared the extent of angiogenesis in control *tg(fli1a:EGFP)^y1^* and *nrp1a^sa1485^ tg(fli1a:EGFP)^y1^* mutant zebrafish at 1 and 3 dpci. GFP-positive vessels were clearly identified within the injured area at 1 and 3 dpci in both wild-type and mutant zebrafish (Fig. 5A,B). However, the *nrp1a^sa1485^* mutation was associated with a significant, 3- to 4-fold reduction in the extent of neovascularization. At 1 dpci, the average number of coronary vessels found within each microscopic field (32,625 μm^2^) of the injury was 13 in wild-type zebrafish compared with three in *nrp1a^sa1485^* mutant zebrafish (*P*=0.0087) (Fig. 5C). At 3 dpci, the average number of newly formed vessels within the injured area per microscopic field of the injury was 19 in wild-type zebrafish compared with six in *nrp1a^sa1485^* mutant zebrafish (*P*=0.0258) (Fig. 5D).

We further examined angiogenesis in cryoinjured hearts from wild-type and *nrp1a^sa1485^* mutant zebrafish at 3 dpci by immunofluorescent staining for the endothelial-specific marker TEK tyrosine kinase, endothelial (Tie2; also known as Tek, https://zfin.org). Use of Tie2 immunostaining as a reliable method to identify neovessels was verified by comparing the number of vessels within the cryoinjury of *tg(fli1a:EGFP)^y1^* zebrafish identified either using Tie2 or GFP immunostaining (data not shown). Similar to the results discussed earlier, obtained with GFP staining of the *tg(fli1a:EGFP)^y1^* transgenic line, the *nrp1a^sa1485^* mutation was associated with a significant reduction in the extent of neovascularization as quantified by Tie2 staining of the neovessels (Fig. S7). At 3 dpci, the average number of coronary vessels found within each microscopic field of the injury was reduced by nearly 50% in *nrp1a^sa1485^* mutants compared with wild-type zebrafish (*P*=0.0058).

### Epicardial activation is inhibited in *nrp1a^sa1485^* hearts

**Fig. 6.**
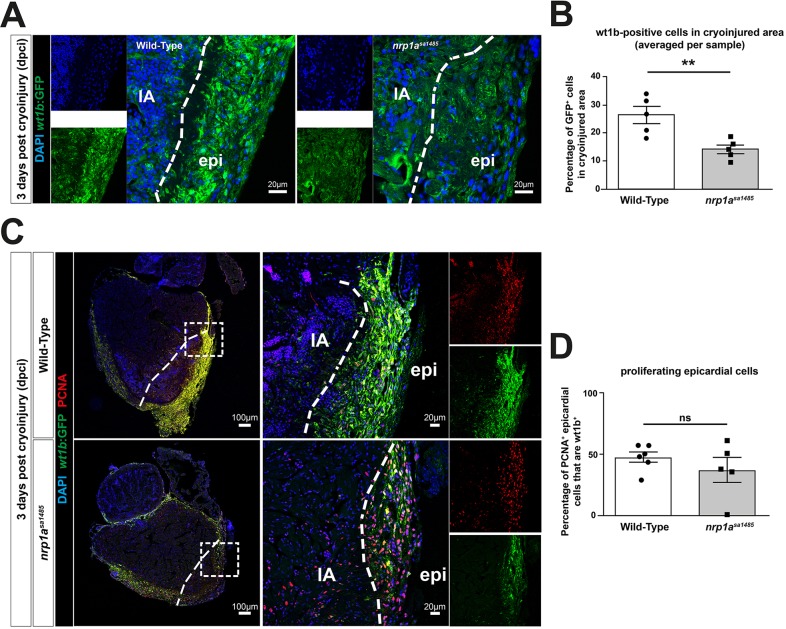
**Epicardial activation is decreased in** ***nrp1a^sa1485^* hearts following cryoinjury.** (A-D) Wild-type and *nrp1a^sa1485^* mutant cryoinjured fish on the *tg(wt1b:EGFP)^li1^* background were analyzed for epicardial activation at 3 dpci by identification of *wt1b*:EGFP-positive cells (A,C), and were also stained with DAPI (blue; A,C) and anti-PCNA antibodies (red; C), as indicated. In A, smaller images represent DAPI staining (blue) and GFP staining (activated epicardium, green) only; larger images are the merged images. In C, boxed area in left panel is enlarged in the middle. Smaller images (right) represent PCNA staining (red) and GFP staining (activated epicardium, green) only; larger images in the middle are the merged images. (B) Quantification of percentages of *wt1b*:EGFP-positive cells adjacent to the area of cryoinjury (indicated by the dashed line in A,C) in wild-type (white bar) and *nrp1a^sa1485^* (gray bar) mutant fish. **P<0.01 (two-tailed *t*-test of *n*=5). (D) Quantification of percentages of cells positive for *wt1b*:EGFP and PCNA in the area of cryoinjury. *P*>0.05 [two-tailed *t*-test of *n*=6 and 5 for wild-type (white bar) and *nrp1a^sa1485^* (gray bar), respectively]. Individual data points (circles for wild type and squares for *nrp1a^sa1485^*) represent percentages in individual hearts, each averaged from counts in two to four different sections covering the injury borders. epi, epicardium; IA, injury area; ns, not significant. White-dotted line delineates injury–epicardial border.

**Fig. 7.**
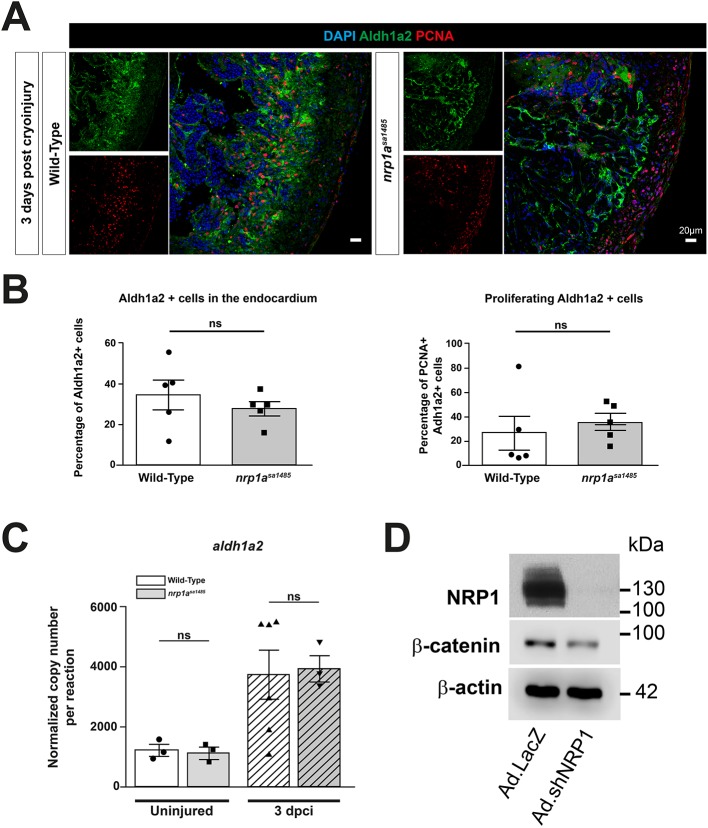
**Aldh1a2 expression is unchanged in *nrp1a^sa1485^* fish following cryoinjury.** (A-D) β-Catenin expression is downregulated in rat epicardial cells. (A) Cryoinjured wild-type and *nrp1a^sa1485^* fish were analyzed for Aldh1a2 expression and were also stained with DAPI and anti-PCNA antibody at 3 dpci. (B) Percentage of Aldh1a2-positive cells in the endocardium of the cryoinjury area (left) and percentage of proliferating endocardial cells (PCNA^+^ and Aldh1a2^+^ cells) (right). *P*=0.43 for Aldh1a2^+^ cells and *P*=0.59 for PCNA^+^ Aldh1a2^+^ cells (two-tailed *t*-test of *n*=5). (C) Absolute qPCR analysis of *aldh1a2* expression was performed on uninjured and 3 dpci ventricles from wild-type and *nrp1a^sa1485^* mutant fish. Bars represent normalized copy number per reaction mean±s.e.m. (one-way ANOVA with Sidak's post hoc test for multiple comparisons of *n*≥3). (D) Rat epicardial cells were cultured *in vitro* and infected with control (Ad.*Lac*Z) and shRNA NRP1 (Ad.shNRP1) adenoviral constructs. Cell lysates immunoblotted for NRP1, β-catenin and β-actin (*n*=2). ns, not significant. Scale bars: 20 µm.

We next addressed whether the delayed heart regeneration caused by loss of functional Nrp1 in *nrp1a^sa1485^* zebrafish could be because of an impact on activation of the epicardium and subsequent epicardial regeneration. Consistent with this possibility, our data showed epicardial upregulation of Nrp1 adjacent to the injured area, indicated by strong colocalization of Nrp1 with Wt1b, a specific marker for epicardial activation, at 3 dpci (Fig. 2C and Fig. S8A), a time coincident with robust epicardium activation during the reparative phase of the regeneration process (González-Rosa et al., 2017). To investigate this hypothesis further, we examined epicardial activation in cryoinjured hearts of wild-type and *nrp1a^sa1485^* mutant *tg(wt1b:EGFP)^li1^* zebrafish. Analysis of hearts at 3 dpci revealed a strong decrease in GFP expression under the control of the *wt1b* promoter in the *nrp1a^sa1485^* mutants compared with wild type (Fig. 6A). Quantification of the percentage of GFP-positive cells within the epicardium covering the cryoinjured area confirmed a marked and significant reduction in the number of GFP-expressing activated epicardial cells in *nrp1a^sa1485^* mutants (14.08% versus 26.4% for *nrp1a^sa1485^* and wild type, respectively; *P*=0.0071) (Fig. 6B). We also investigated whether loss of functional *nrp1a* impaired the proliferation of activated epicardial cells, using Proliferating Cell Nuclear Antigen (PCNA) staining (Fig. 6C). *Nrp1a^sa1485^* hearts showed no statistically significant reduction in the proportion of proliferating epicardial cells expressing *wt1b* compared with wild-type hearts (37.25% versus 47.68% for *nrp1a^sa1485^* and wild type, respectively; *P*=0.34) (Fig. 6D). Given that WT1 is known to regulate epicardial EMT through the retinoic acid signaling pathway (von Gise et al., 2011), we investigated *aldh1a2* gene and protein expression in 3-dpci hearts of wild-type and *nrp1a^sa1485^* fish (Fig. 7). *aldh1a2* was upregulated following cryoinjury, but there was no significant difference between *aldh1a2* gene expression in wild-type and *nrp1a^sa1485^* hearts (*P*=0.99 for uninjured, *P*=0.97 for 3 dpci) (Fig. 7C). We also stained Aldh1a2-positive cells in sections of 3-dpci heart (Fig. 7A) and quantified these cells in the endocardium, as well as the number of proliferating (PCNA-positive) Aldh1a2-positive cells (Fig. 7B). Our results revealed no significant difference in Aldh1a2-positive cells between wild-type and *nrp1a^sa1485^* mutant hearts (*P*=0.43 Aldh1a2^+^ cells, *P*=0.59 PCNA+ Aldh1a2^+^ cells) (Fig. 7B).

The gene expression of other known EMT effectors, such as smooth muscle actin (*acta2*), *T-box 18* (*tbx18*), transforming growth factor beta receptor 1 a (*tgfbr1a*), and Fgf receptor 2 and 4 (*fgfr2* and *fgfr4*), was unchanged in *nrp1a^sa1485^* compared with wild-type hearts (data not shown). Thus, *nrp1b* might compensate for *nrp1a* inactivation in these pathways. In addition, the *nrp1a^sa1485^* mutation did not result in an upregulation of the other neuropilin isoforms following cryoinjury (wild type versus *nrp1a^sa1485^* for *nrp1b*, *P=*0.4674; *nrp2a*, *P=*0.9026; and *nrp2b*, *P=*0.051) (Fig. S9).

Another known epicardial signaling effector downstream of WT1 is β-catenin (von Gise et al., 2011). To investigate the possibility that NRP1 is implicated in the regulation of β-catenin expression, we used the rat epicardial cell line described by Wada et al. (2003) and examined the effect in these cells of an adenovirus encoding a shRNA targeting NRP1 or a control adenovirus. We found that, after 48 h infection, there was a downregulation of β-catenin in NRP1-depleted cells compared with controls (Fig. 7D).

Additionally, we examined whether cardiomyocyte proliferation was affected in *nrp1a^sa1485^* mutant hearts by determining the number of Myocyte Enhancer Factor 2C (Mef2C)-positive cells that were also PCNA positive. We counted double positive cells both at the border and within the injured area and found that there was no significant difference in the number of proliferating cardiomyocytes (*P*=0.065 and *P*=0.54, respectively) (Fig. S10A,B).

### Epicardial expansion and activation of cryoinjury-induced *nrp1a^sa1485^* heart explants is impaired

**Fig. 8.**
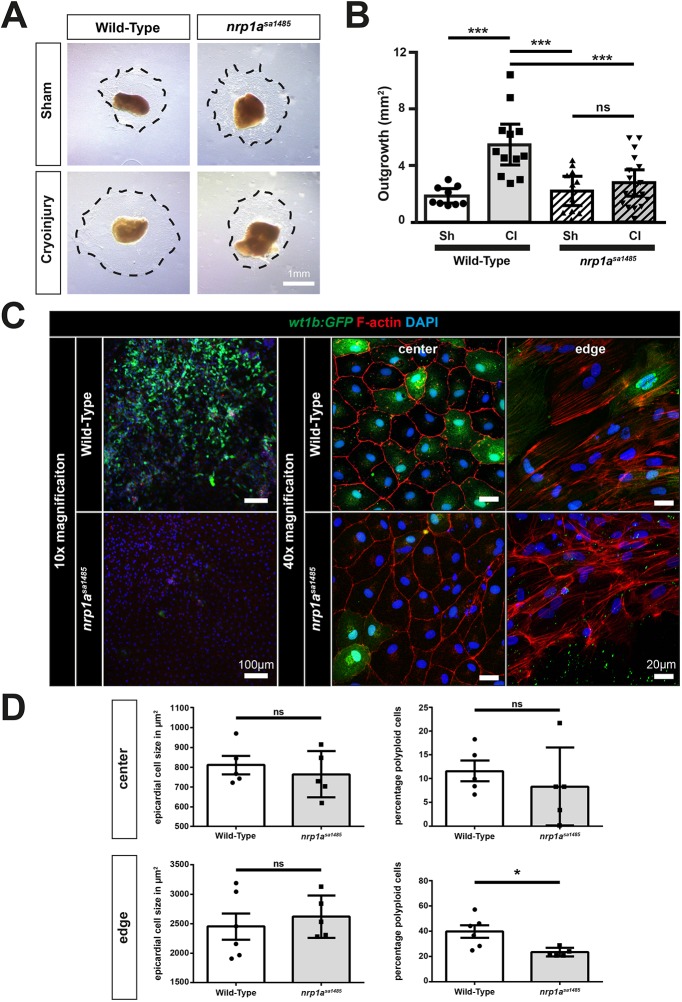
**Epicardial cryoinjury-induced expansion and activation are impaired in *nrp1a^sa1485^* mutants.** (A-D) The apices of wild-type and *nrp1a^sa1485^* zebrafish ventricles were collected 5 days post sham surgery or cryoinjury and cultured on fibrin gels for 7 days. (A) Epicardial cells migrated into the fibrin gels (dotted black lines). (B) Epicardial outgrowths were measured for each condition (sh, sham-operated and CI, cryoinjured hearts) after 7 days culture. Data are mean outgrowth area (mm^2^)±s.e.m. ***P<0.001 (one-way ANOVA with Sidak's post hoc test for multiple comparisons of *n*>9). (C) Epicardial explant recovered from wild-type and *nrp1a^sa1485^ tg(wt1b:EGFP)^li1^* cryoinjured fish at 5 dpci were left to grow on fibrin gels for 7 days and stained for GFP. GFP fluorescence was observed at 10× (left column) and 40× magnification at the center and the periphery of the explants (middle and right columns, respectively). (D) Cell size (left) and ploidy (right) were quantified both at the center (top row) and at the edge (bottom row) of the explant. Data are expressed as percentage of cells per field of view ±s.e.m. Each *n* represents an average of 3 fields of view per explant (two-tailed *t*-test of *n*≥5); *P<0.05. ns, not significant. Scale bars: 1 mm in A, 20 µm in C (right); 100 µm in C (left).

We next examined the role of Nrp1 in epicardial activation in an *ex vivo* heart explant model (Kim et al., 2012). Immunofluorescent staining of Nrp1 in explants of wild-type ventricular apexes collected at 5 dpci and cultured *in vitro* for 7 days showed perinuclear, cytoplasmic and membrane localization (Fig. S8B). Epicardial culture from *tg(wt1b:EGFP)^li1^* ventricles showed that *Wt1b*:EGFP expression was variable in these explants and was strongly expressed by a subpopulation of explanted epicardial cells (Fig. 8C, top row).

*wt1b*:EGFP expression was increased in explant outgrowths from cryoinjured compared with those from control, sham-operated hearts (Fig. S11A,C) and, similar to resected hearts (Kim et al., 2012), explants from wild-type cryoinjured hearts generated greater outgrowth compared with those from wild-type sham-operated hearts (*P*=0.0001; Fig. 8A,B). We next assessed the role of Nrp1a in injury-induced epicardial activation using epicardial explants from wild-type and *nrp1a^sa1485^ tg(wt1b:EGFP)^li1^* zebrafish. Epicardial outgrowths of cryoinjury-induced *nrp1a^sa1485^* heart explants were markedly impaired compared with wild-type explants (*P*=0.0009) (Fig. 8A,B) and we observed no significant difference between the outgrowths of either wild-type or *nrp1a^sa1485^* mutant sham-operated hearts (*P=*0.93 and *P=*0.99, respectively). Furthermore, *nrp1a^sa1485^* explants showed a marked decrease in GFP expression compared with wild-type hearts, both at the edge of the explant and within the region closest to the heart (Fig. 8C), providing further support for a loss of epicardial activation in the *nrp1a^sa1485^* mutants compared with wild-type hearts. This confirmed our observations of reduced GFP-positive epicardial cells in sections from cryoinjured *nrp1a^sa1485^ tg(wt1b:EGFP)^li1^* zebrafish hearts (Fig. 6A,B). The less marked effect of the *nrp1a^sa1485^* mutant on epicardial activation observed in heart sections compared with cultured epicardial explants, might reflect compensatory effects of other cell types (such as fibroblasts or immune cells) secreting paracrine factors to drive epicardial activation and partially rescue the *nrp1a* mutant phenotype *in vivo*, which are lacking in epicardial cultures. Cao et al. (2017) recently showed that transient hypertrophy and polyploidization have an important role in epicardial regeneration following induced cell death in the zebrafish heart. To investigate whether changes in cell size or ploidy had a role in the effect of the *nrp1a^sa1485^* mutant on injury-induced epicardial regeneration, we also analyzed the size and ploidy of the cells at the center and at the edge of the ventricular explant (Fig. 8D). There was no significant difference in cell size, neither at the center (*P*=0.53) nor at the edge of the explant (*P*=0.57), but we observed a reduction in the number of polyploid cells at the edge of the explant in the *nrp1a^sa1485^* mutants compared with wild-type hearts (*P*=0.016), whereas there was no difference in the center of the explant (*P*=0.46).

## DISCUSSION

Epicardial activation and angiogenesis are processes essential for zebrafish heart regeneration following injury. During these processes, revascularization and injury-induced EMT are driven by Vegf, Fgf and Pdgf (Chablais and Jazwinska, 2012; Kim et al., 2010; Lepilina et al., 2006), which are all ligands for the cell surface receptor Nrp1. Although it is known that NRPs are essential for angiogenesis and are increasingly implicated in EMT in other contexts in mammals (Adham et al., 2014; Chu et al., 2014; Kawasaki et al., 1999), their role has not previously been characterized in zebrafish heart regeneration. Here, we show for the first time that *nrp1* and *nrp2* are upregulated in response to cardiac injury and that *nrp1a* has a role in revascularization and epicardial activation and migration, processes that are essential for the regeneration of the zebrafish heart.

*Nrp1a*, *nrp1b*, *nrp2a* and *nrp2b* mRNAs were all strongly upregulated in the zebrafish heart 1-3 days after cryoinjury, coinciding with the time of epicardial activation, which occurs very early following cardiac injury (Cao and Cao, 2018). Increased protein expression of both Nrp1 isoforms also occurred 3-14 days following cryoinjury, further supporting the conclusion that Nrp1 is upregulated in the early regenerating heart. Our results also revealed a striking spatiotemporal upregulation of the nrp family isoforms. Specifically, *nrp2a* was strongly upregulated in the endocardium (1 dpci) and in the epicardium proximal to the injury (3 dpci), whereas *nrp1a* was strongly upregulated in the same regions at 1, 3 and 14 dpci. These findings support a sustained role for these isoforms in heart regeneration, particularly in the epicardial activation phase, which occurs during the first 3-7 days of regeneration. Although the lack of suitable reagents made further detailed studies of Nrp2 problematic, a role for Nrp1 in epicardial activation in response to heart injury was further supported by immunofluorescent staining demonstrating colocalization of Nrp1 with both endogenous Wt1 and with EGFP under the control of the *wt1b* promoter. This is an embryonic gene that is upregulated following cardiac injury and is an activated epicardium marker (Peralta et al., 2014; von Gise et al., 2011).

Analysis of mutant *nrp1a^sa1485^* fish lacking expression of full-length Nrp1a provided direct evidence that Nrp1a is required for zebrafish heart regeneration. *Nrp1a^sa1485^* fish displayed no morphological or pathological phenotype. It was previously reported that knockdown of *nrp1* using morpholino oligomers produces a lethal phenotype in zebrafish embryos (Martyn and Schulte-Merker, 2004). The absence of embryonic lethality in *nrp1a^sa1485^* fish compared with *nrp1a* morpholino knockdown probably reflects redundancy because of adaptive mechanisms relying on compensation by *nrp1b* and also suggest morpholino off-target effects in *nrp1a* morphants (Kok et al., 2015). The genetic robustness of the *nrp1a^sa1485^* mutant fish could also be the result of the allele displaying mutant mRNA decay (El-Brolosy et al., 2019), although we could not detect any compensative upregulation of the other nrp-related genes. Following cardiac damage, *nrp1a^sa1485^* mutants exhibited a significantly reduced regenerative response compared with wild-type controls. The importance of *nrp1a* for heart regeneration was demonstrated by the delayed and incomplete removal of fibrin deposits essential for the scar resolution process in *nrp1a^sa1485^* mutant fish. Given that myocardial proliferation was not significantly affected in *nrp1a^sa1485^* mutant fish, delayed wound closure in *nrp1a^sa1485^* fish probably indicates a failure of the myocardium to migrate efficiently towards the subepicardial layer after cryoinjury. Together, these findings provide strong evidence that *nrp1*a is required for zebrafish heart regeneration following cryoinjury. Given that we examined the loss of only the *nrp1a* isoform, because of the anticipated embryonic lethality of a double *nrp1a* and *nrp1b* knockout, it is possible that Nrp1 loss would have an even more prominent role in heart regeneration in species that did not undergo genome duplication. Furthermore, our data also showed epicardial expression of *nrp2a* and *nrp2b*, indicating a possible role of Nrp2 isoforms in epicardial activation and heart regeneration, something that warrants further investigation.

Activation of the epicardium and subsequent regeneration involves multiple cellular processes, including cell migration, proliferation and EMT. It is well established that NRP1 modulates cell migration in diverse mammalian cell types (Evans et al., 2011; Pellet-Many et al., 2008; Wang et al., 2003). The conclusion that *nrp1a* is important for zebrafish epicardial migration is supported by our finding that *ex vivo* outgrowth from epicardial explants of *nrp1a^sa1485^* hearts was also impaired. Furthermore, we observed a reduction in polyploidization of explanted epicardial cells in *nrp1a^sa1485^* hearts, a process that was recently implicated as an important mechanism underlying epicardial regeneration following induced cell death in zebrafish (Cao et al., 2017). By contrast, we observed no effect on epicardial cell proliferation in cryoinjured *nrp1a^sa1485^* hearts, indicating that *nrp1a* is not crucial for proliferation, in line with studies of NRP1 function in primary mammalian cells. However, we cannot preclude the possibility that one of the other Nrp isoforms has a role in epicardial proliferation.

Reduced *in vitro* expansion of *nrp1a*-deficient epicardial cells could have been the result of impaired detection of cellular cues promoting migration. Consistent with this possibility, we observed upregulation of Nrp isoform expression concomitant with increased expression of *tgfb*, *pdgfab*, *vegfc* and the receptor *pdgfrb*, chemotactic factors and receptors implicated in zebrafish heart regeneration, and also shown to act as ligands and co-receptors for NRP1 in mediating mammalian cellular functions. Interestingly, using the *(wt1b:EGFP)^li1^* transgenic fish line, we also noted that the *nrp1a^sa1485^* epicardial cells failed to re-express the *wt1b* embryonic marker *in vitro* as well as *in vivo*. Previously, González-Rosa et al. (2012) demonstrated the importance of *wt1b*:EGFP^+^ epicardial derived cells (EPDCs) in the regeneration process that gives rise to perivascular fibroblasts and myofibroblasts and that these cells also participated in the regeneration process by secreting essential proangiogenic paracrine factors. Therefore, the decreased number of GFP-expressing (and, therefore, *wt1b^+^*) EPDCs in the *nrp1a^sa1485^* hearts probably explains their delayed regeneration compared with wild-type hearts. WT1 regulates epicardial EMT through β-catenin and retinoic acid signaling pathways in mice (von Gise et al., 2011) and interruption of Wnt/β-catenin signaling in epicardial cells disrupts EMT and compromises cardiac function after acute cardiac injury (Duan et al., 2012). Although we observed an upregulation of *aldh1a2* gene and protein expression in cryoinjured hearts in both *nrp1a^sa1485^* wild-type fish, there was no significant difference in either gene or protein expression at 3 dpci, indicating that Nrp1 functions in epicardial regeneration via Aldh1a2-independent pathways downstream of Wt1b.

Our study also revealed Nrp1 upregulation following cardiac damage by the activated endocardium, which undergoes endothelial to mesenchymal transition (endoMT) in response to injury (Kikuchi et al., 2011), by the neovasculature and by some subepicardial cardiomyocytes known to be a primary source of new myocardium (Kikuchi et al., 2010). Following injury, these cells acquire a migratory phenotype to contribute to the regenerative processes in the heart. It is probable that the *nrp1a^sa1485^* endocardium is less able to perform this function, further contributing to the overall observed delay in regeneration.

Nrp1 has an essential role in angiogenesis in mammalian and zebrafish development, and is required in postnatal and adult angiogenic processes (Lee et al., 2002; Soker et al., 1998). Marín-Juez et al. recently reported transient upregulation of *vegfaa* at 1 dpci, with a return to baseline expression by 3 dpci, and showed an important role for *vegfaa* in inducing rapid early revascularization of the injured heart (Marín-Juez et al., 2016). Based on RT-qPCR, our data showed a trend towards increased *vegfaa* expression at 1 dpci, although this was not statistically significant, unlike the concomitant changes in *nrp1*. Similarly to its major endothelial ligand *vegfaa*, the main Nrp1 co-receptor *kdrl* was also not significantly upregulated. However, we observed revascularization of the injured area as early as 1 dpci, in line with previous findings (Marín-Juez et al., 2016). These neovessels also expressed Nrp1, and studies in *nrp1a^sa1485^* mutants co-expressing *fli1a*:*EGFP^y1^* demonstrated a role for *nrp1a* in the revascularization of the cryoinjured area. As expected, the loss of Nrp1a reduced the number of neovessels in the regenerating heart of *nrp1a^sa1485^* fish compared with their wild-type counterparts. Although our findings are consistent with a role for Nrp1 in mediating Vegfaa-driven angiogenesis in the regenerating heart, recent findings indicate that the role of NRP1 in developmental angiogenesis might be largely independent of VEGF, because NRP1 mutations that prevent VEGFA binding impair postnatal angiogenesis but are compatible with normal embryonic development (Fantin et al., 2015; Fantin et al., 2014). Therefore, it is plausible that the angiogenic role of Nrp1 in revascularization of the regenerating zebrafish heart is also mediated via binding of other ligands to Nrp1 (Ball et al., 2010; Pellet-Many et al., 2011; West et al., 2005).

This study establishes a novel role for Nrp1 in epicardial activation and angiogenesis during zebrafish heart regeneration following injury. Further work to elucidate the extracellular ligands for Nrp1 in epicardial and endothelial cells and the signaling pathways that mediate its role further downstream will shed new light on the mechanisms involved in epicardial activation in heart regeneration.

## MATERIALS AND METHODS

### Zebrafish husbandry and cryoinjury

Procedures were performed in accordance with the Animals (Scientific Procedures) Act 1986, and husbandry was regulated by the central University College London fish facility. Cryoinjury procedure was carried out as described by González-Rosa and Mercader (2012), and more details are provided in the supplementary Materials and Methods.

### RT-qPCR

Ventricles from corresponding time-points and treatments were pooled for RNA extraction using the RNeasy Mini Kit (Qiagen). RNA was reverse transcribed using the QuantiTect^®^ Reverse Transcription Kit (Qiagen). All primers (details are described in Table S1) and standards were purchased from qStandard^©^ and absolute qPCR was performed by qStandard^©^. More details are provided in the supplementary Materials and Methods.

### Histological procedures

*In situ* hybridization, immunofluorescence and AFOG procedures are described in the supplementary Materials and Methods.

### Fibrin gel heart explants

*In vitro* epicardial cell outgrowth experiments were performed as previously described (Kim et al., 2012). The apices of cryoinjured and sham-operated zebrafish hearts were isolated 5 days post surgery and placed firmly on set fibrin gel matrices, ensuring epicardial surface contact with the gel. Medium was changed every 2 days and cells were cultured for 7 days before harvesting epicardial outgrowths for protein extraction or immunofluorescence imaging. More details are provided in the supplementary Materials and Methods.

### Immunoblotting

Epicardial cell lysates were obtained from a single well of a six-well plate and zebrafish heart lysates from a minimum of three ventricles pooled together. Protein contents were separated using SDS-PAGE, electro-transferred to PVDF membranes and blocked in 5% milk in PBS containing 0.1% Tween-20 (PBST). Membranes were incubated with primary antibodies overnight at 4°C. Western blot band densities were normalised to GAPDH and analysed using ImageJ. Blots were washed with PBST and proteins detected using HRP-conjugated secondary antibodies and ECL detection with Hyperfilm. More details about protein extraction, antibodies and immunoblotting protocol are provided in the supplementary Materials and Methods.

### Recombinant adenovirus generation

Rat-specific NRP1 shRNA construct was generated as previously described (Pellet-Many et al., 2015), and adenovirus generation and reagents are detailed in the supplementary Materials and Methods.

### Epicardial cell NRP1 knockdown

Rat epicardial cells were a generous gift from Dr Nicola Smart (Department of Physiology, Anatomy and Genetics, University of Oxford, UK) and are described in Wada et al. (2003). Cells were infected with adenovirus at a multiple of infection (MOI) of 100 for 24 hours before protein extraction and subsequent immunoblotting as described above. See supplementary Materials and Methods for more details.

### Statistical analysis

All results are presented as mean±s.e.m. Experimental repeat *n* values are indicated as individual data points in graphs or are specified in figure legends. When samples were pooled to produce 1 *n* (e.g. for qPCR) or several sections per tissue were analyzed, this is indicated in the figure legends or the main text. All data were visualized and analyzed using Graphpad prism 6.0 software. All data were first tested for normal distribution using histograms and the D'Agostino–Pearson Omnibus test. Comparisons of more than two groups (e.g. AFOG cryoinjury area and qPCR data) were analyzed for statistical significance using one-way ANOVA with preselected pairs and the Sidak's post hoc test for multiple comparisons and, for overall effect, a two-way ANOVA with Tukey post-test was conducted. Student's unpaired *t*-tests were applied to all other data sets, (i.e. for data sets comparing only two groups). The Mann–Whitney test was applied for comparisons of two groups if data were not normally distributed. Statistical significance values are indicated in figure legends and in the main text. Data were considered significant at *P*<0.05. All immunostaining data were analyzed by a blinded investigator.

## Supplementary Material

Supplementary information
