# Supplementary material for: Neuropilin 1 mediates epicardial activation and revascularization in the regenerating zebrafish heart
Source: Development. 2019 Jul 2;146(13):dev174482. doi: 10.1242/dev.174482 (PMC6633600; doi:10.1242/dev.174482)
Supplement: Supplementary information [file develop-146-174482-s1.pdf]

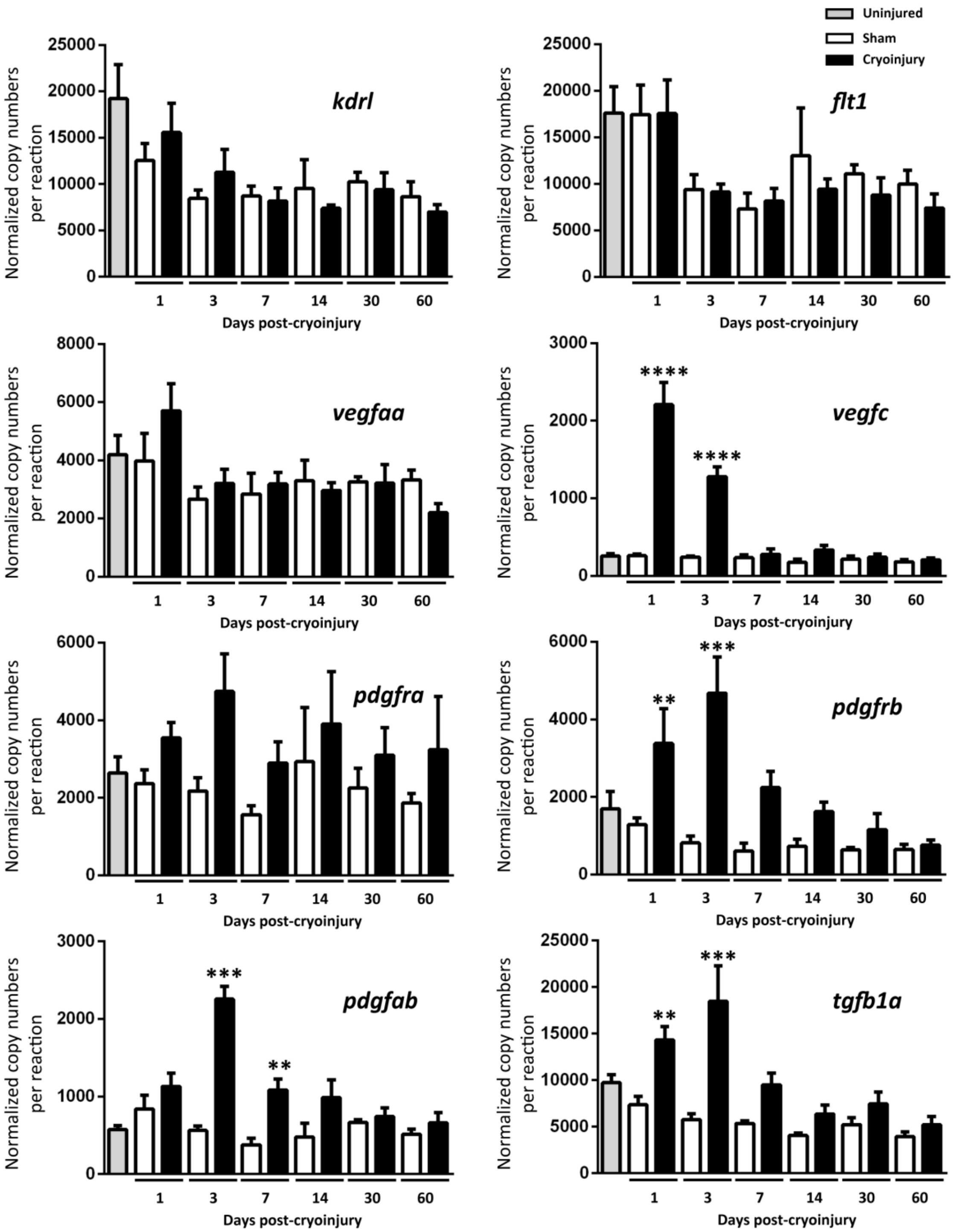

Figure S1 Gene expression of vascular endothelial growth factors (*vegf*), platelet-derived growth factor (*pdgf*) and transforming growth factor (*tgf*) and their receptors in adult zebrafish cryoinjured ventricles.

Absolute quantitative PCR analysis 1, 3, 7, 14, 30, and 60 days following cryoinjury or sham surgery of zebrafish ventricles. Basal expression was evaluated in uninjured hearts. *Vegf* receptors *kdrl* and *flt1* and *vegf* isoforms (*vegfaa* and *vegfc*) mRNA levels

Development • Supplementary information

were assessed to evaluate expression of genes associated with angiogenesis. *Pdgf* receptors *pdgfra* (*pdgfr*α) and *pdgfrb* (*pdgfr*β), additional to *pdgf* (*pdgfab*) and *tgf* (*tgfb1a*) gene expression were measured to evaluate expression of genes associated epithelial-to-mesenchymal transition. Bars represent means of normalized copy numbers per reaction ± S.E.M, ***p*<0.01, ****p*<0.005, *****p*<0.001 (one-way ANOVA with Sidak's *post hoc* test for multiple comparisons of *n* = 4-5 with each *n* being a pool of 5 ventricles).

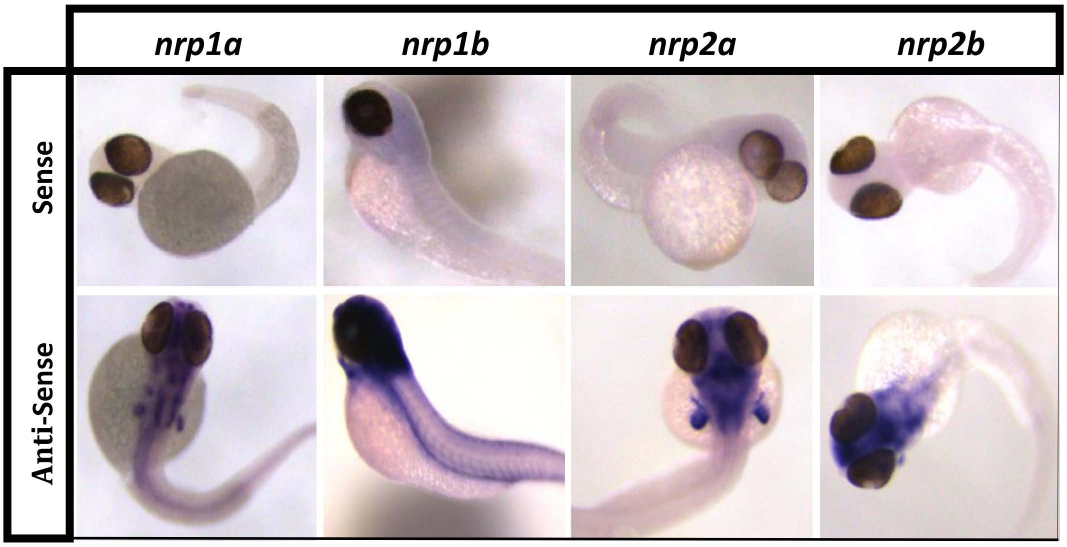

Figure S2 *nrp* riboprobes validation.

In situ hybridization of TraNac transgenic zebrafish embryos 48 hours post fertilization (hpf) with *nrp* sense riboprobes **(upper row)** and *nrp* anti-sense riboprobes **(lower row)**. Anti-sense riboprobes differential staining patterns were compared to previous reports (43) to confirm specific *nrp* isoform detection. All neuropilin isoforms are observed in the brain with additional differential expression patterns observed between different isoforms. *Nrp1a* is observed in the fin buds and otic vesicles, *nrp1b* is expressed in the dorsal aorta and intersegmental vessels, *nrp2a* is observed in the hind brain and fin buds, whereas *nrp2b* is largely restricted to the brain and hind brain, $n \geq 8$.

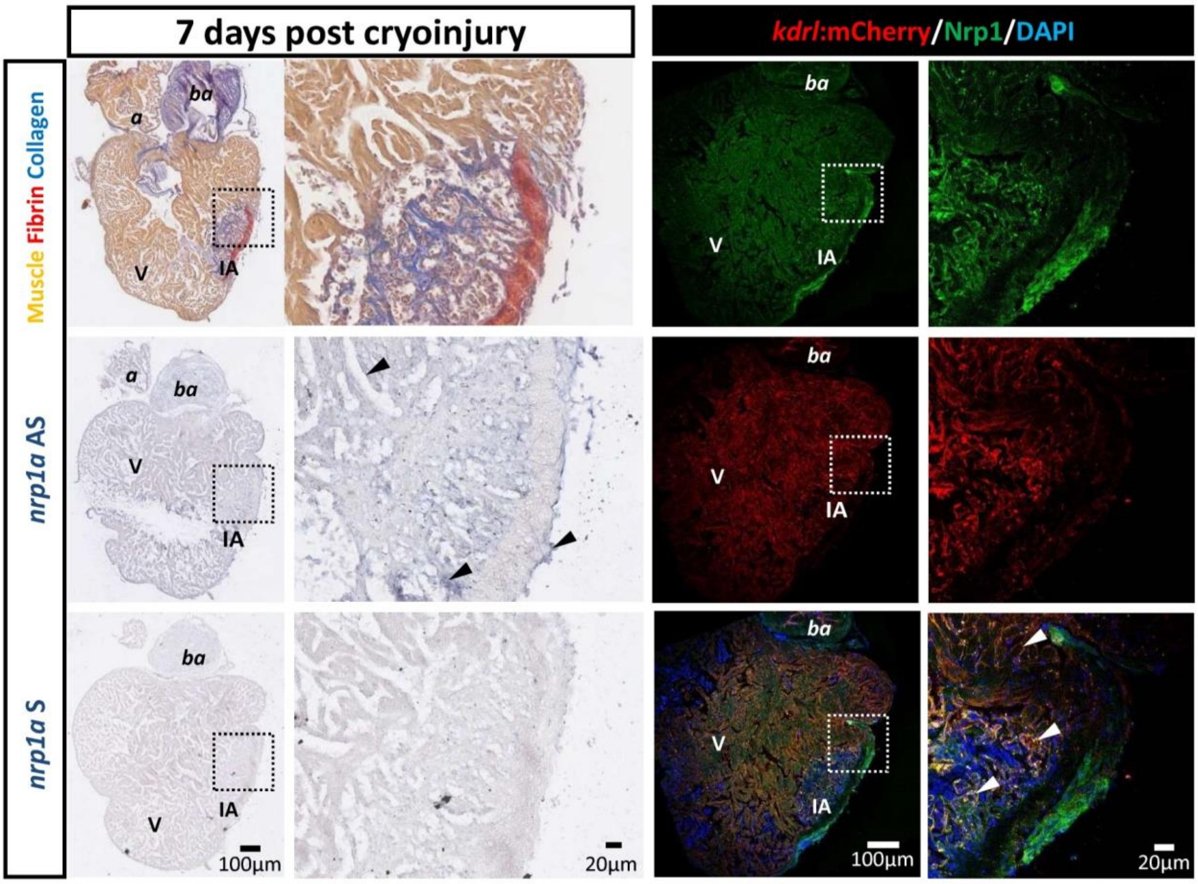

Figure S3 Nrp1 is expressed by the endocardium.

AFOG staining **(upper left panels)**, *in situ* hybridization (ISH) **(middle and lower left panels)** and immunofluorescence **(two right columns)** of *tg(kdrl:mCherry)*^{s896} zebrafish heart 7 days post cryoinjury (dpci). AFOG staining gives reference to cryoinjury location and tissue composition. ISH of *nrp1a* anti-sense riboprobe (*nrp1a* AS) **(middle left panels)** and negative control sense (*nrp1a* S) riboprobe **(lower left panels)**, signal is observed as a dark blue stain within the section, black arrows indicate mRNA expression. *kdrl* expression by viable endocardium is immunolabeled with anti-mCherry antibody (red) **(middle right panels)** and Nrp1-expressing cells are labelled in green **(upper right panels)**. Overlay of the two colors is displayed with DAPI nuclei staining **(lower right panels)**. White arrows indicate regions of colocalization. Dotted boxes highlight magnified regions. V– ventricle, *ba*– *bulbus arteriosus*, *a*– atrium, IA– injured area (n = 2).

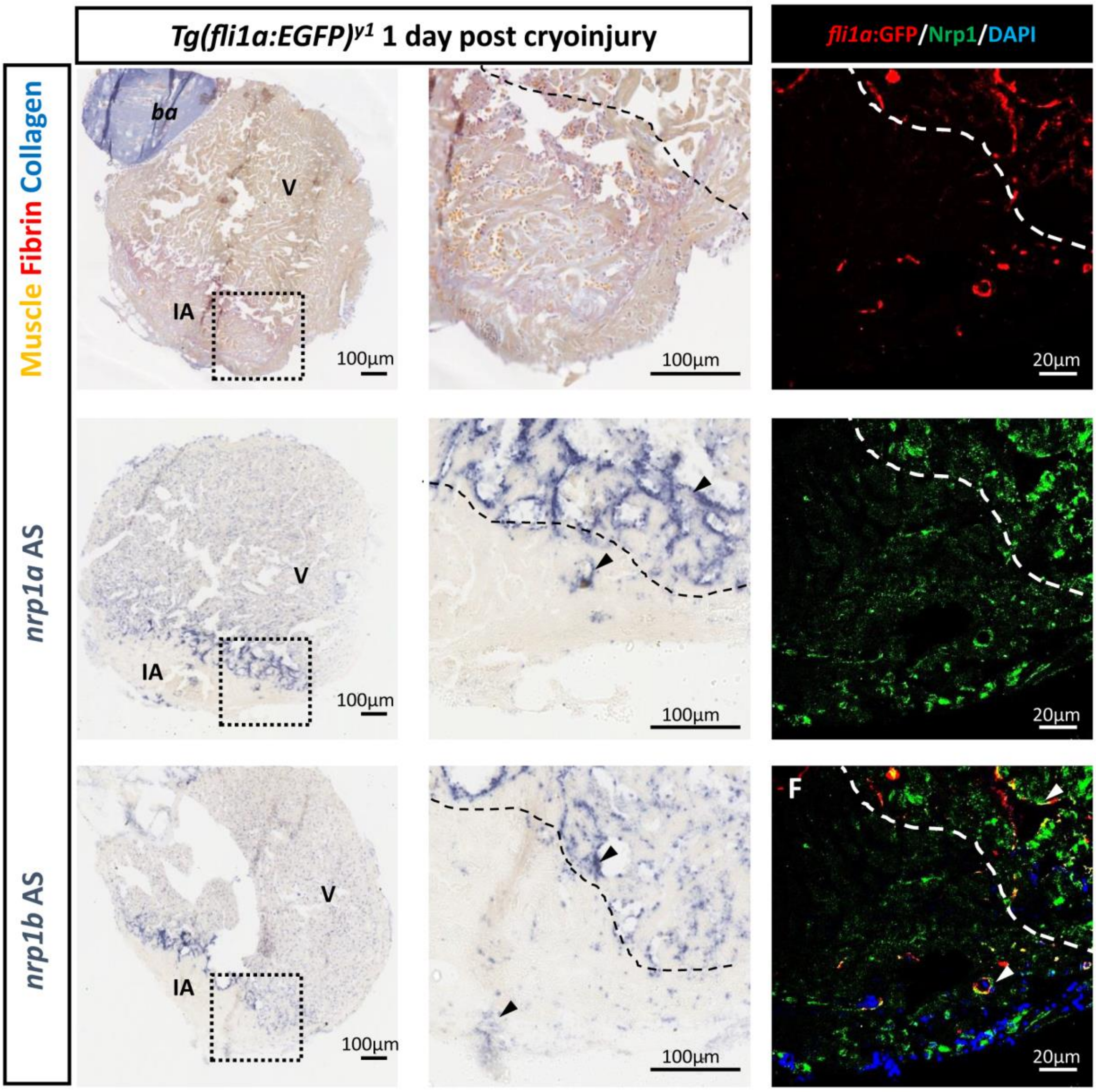

Figure S4 Nrp1 is expressed by early neovasculature in the cryoinjured lesion of *tg(fli1a:EGFP)*^{y1} zebrafish heart 1 day post cryoinjury.

AFOG staining **(upper left)** identifies cryoinjured lesion. *Nrp1a* **(middle left)** and *nrp1b* **(lower left)** mRNA localization is detected with *in situ* hybridization, black arrows indicate mRNA expression within the injury and at the injury/healthy myocardium border. Immunofluorescence imaging was used to locate *fli1a:*EGFP positive cells expressed by viable endothelium and endocardium (red) **(upper right)** and Nrp1-expressing cells (green) **(middle right)**. Overlay of the two colors are displayed with DAPI nuclei staining **(lower right pane)**, white arrows indicate areas of colocalization. Dotted boxes highlight magnified regions. Dashed lines define injury interface. V– ventricle, *ba*– *bulbus arteriosus,* IA– injured area, *n* = 3.

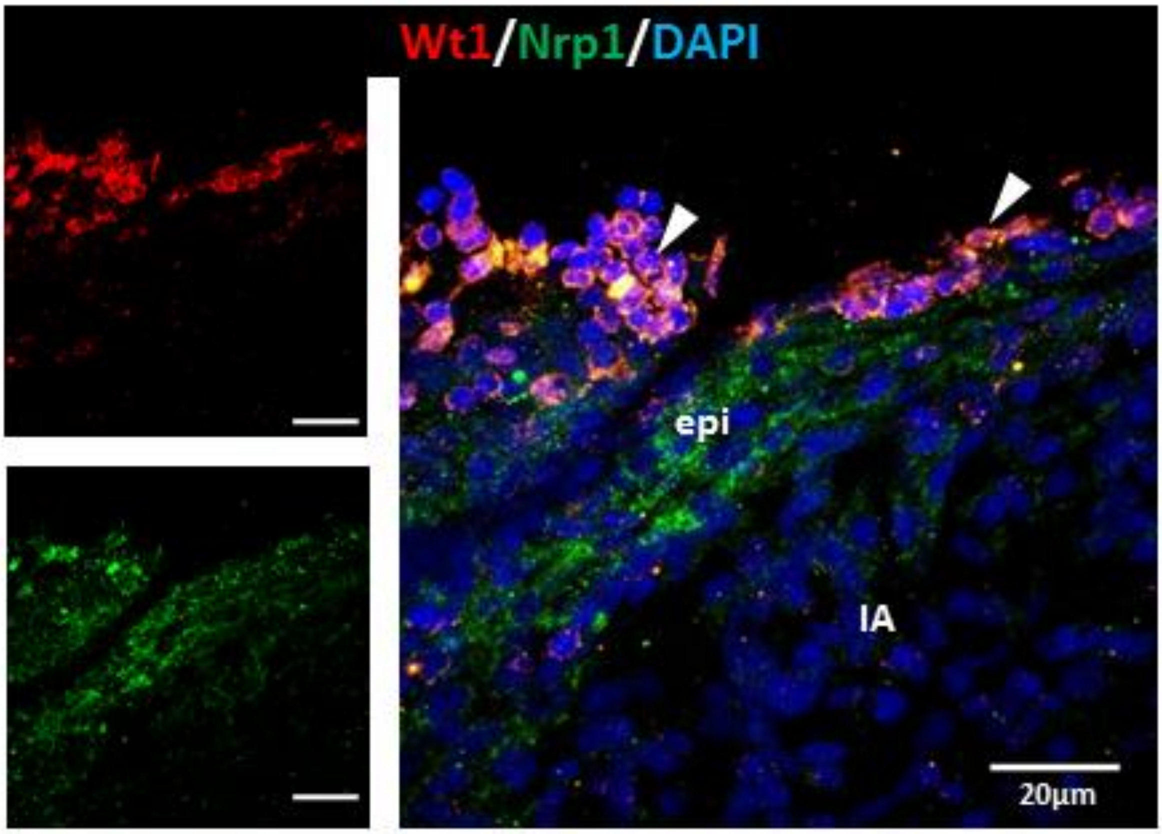

Figure S5 Wt1-positive epicardial cells express Nrp1.

Immunofluorescence of Wild-Type adult zebrafish heart 3 days post cryoinjury (dpci). Activated epicardial cells were identified with Wt1 antibody (red) and assessed for Nrp1 expression (green). White arrows indicate regions of colocalization. Scale bars = 20µm. IA– injured area, epi– epicardium (*n* = 2).

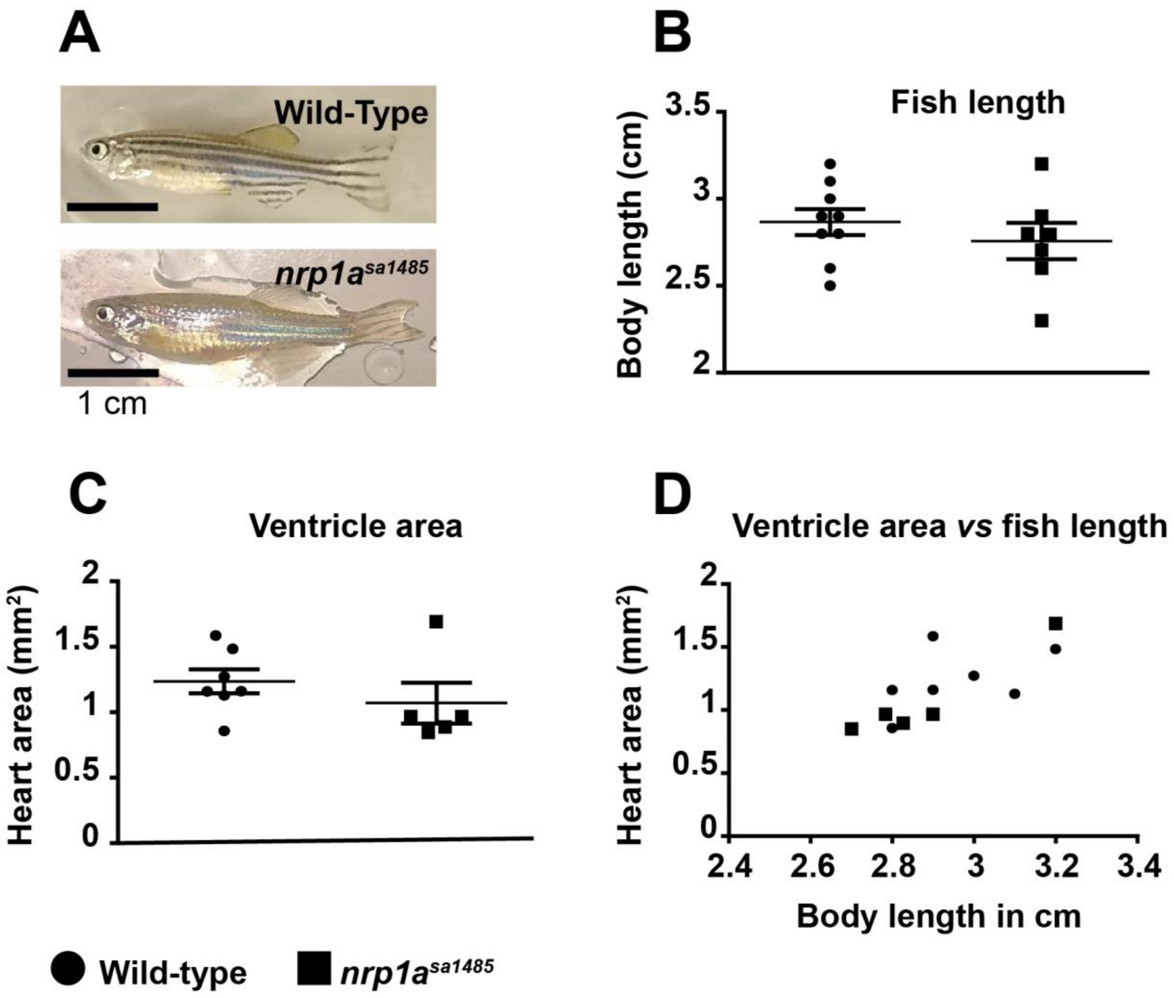

Figure S6 *nrp1a^{sa1485}* mutant fish characterization

(A) Representative picture of Wild-Type (upper panel) and *nrp1a^{sa1485}* mutant zebrafish (lower panel), scale bar 1 cm. The body length **(B)**, and heart size **(C)** of age matched Wild-Type (black dots) and *nrp1a^{sa1485}* mutant (black squares) zebrafish were measured and compared (two-tailed *t*-test of $n \geq 5$, $p>0.05$). **(D)** Scatter graph representing the values of body length to heart size ratio. Values are displayed as individual measurements of fish indicated as black dots (Wild-Type) or squares (*nrp1a^{sa1485}*).

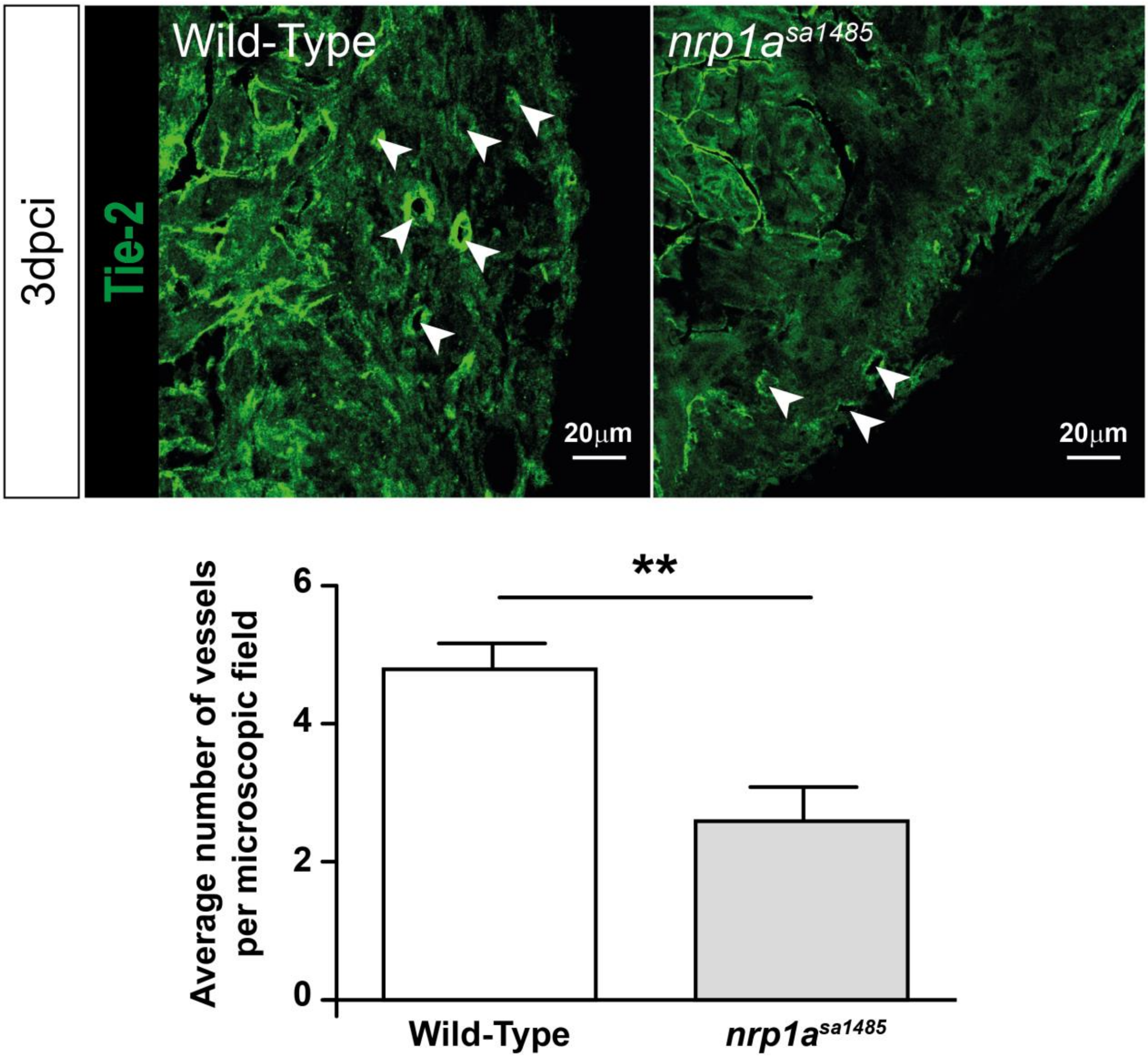

Figure S7 Neovascularization of the cryoinjured area is impaired in *nrp1a*^{sa1485} fish.

Tie2 immunostaining of Wild-Type (top left) and *nrp1a*^{sa1485} (top right) hearts 3 days post cryoinjury. Bars represent average numbers of newly formed vessels per microscopic field (32625μm^2) within the injured area ± S.E.M, samples were quantified at least at 3 levels across the whole hearts (two-tailed *t*-test *of n* = 6, ** *p*<0.01).

Development • Supplementary information

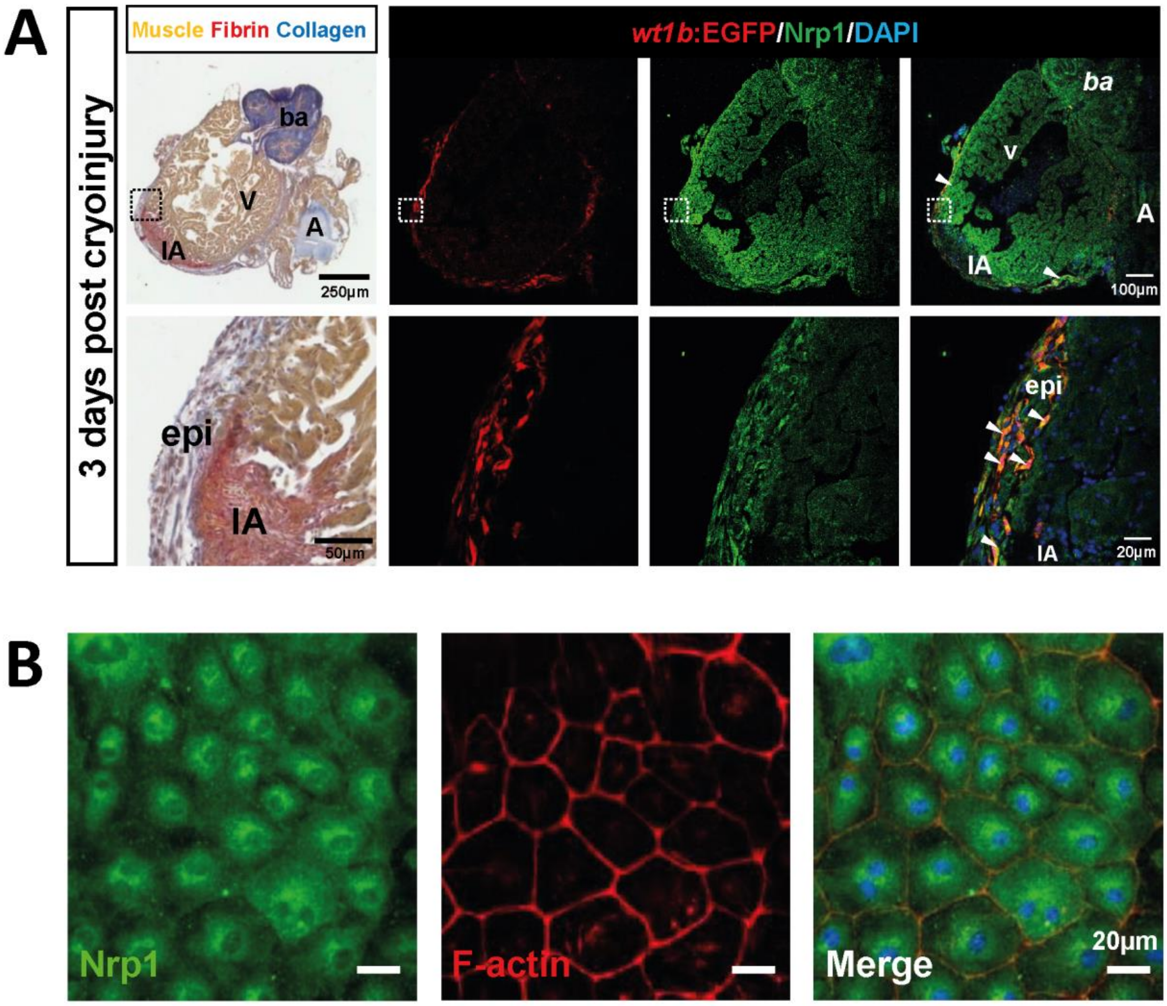

Figure S8 Epicardial cells express Nrp1 *in vivo* and *in vitro.*

(A) Serial sections of *tg(wt1b:EGFP)*^{li1} adult zebrafish heart 3 dpci stained with AFOG for lesion location and immunostained for GFP (red), Nrp1 (green) and DAPI (right panels). Dotted boxes indicate magnified region in panels below. White arrows identify cells co-expressing GFP and Nrp1. V– ventricle, *ba– bulbus arteriosus*, A– atrium, IA- injured area, epi- epicardium (*n* = 3). **(B)** Wild-Type ventricle apices were collected 5 dpci and cultured *in vitro* for 7 days and epicardial outgrowths immunostained for Nrp1. All epicardial cells of the explant were Nrp1 positive. Phalloidin conjugated to Alexa-555 was used to identify F-actin and highlight cellular tight junctions and DAPI staining applied to located individual cells. Scale bar 20 µm (*n* = 4).

Development • Supplementary information

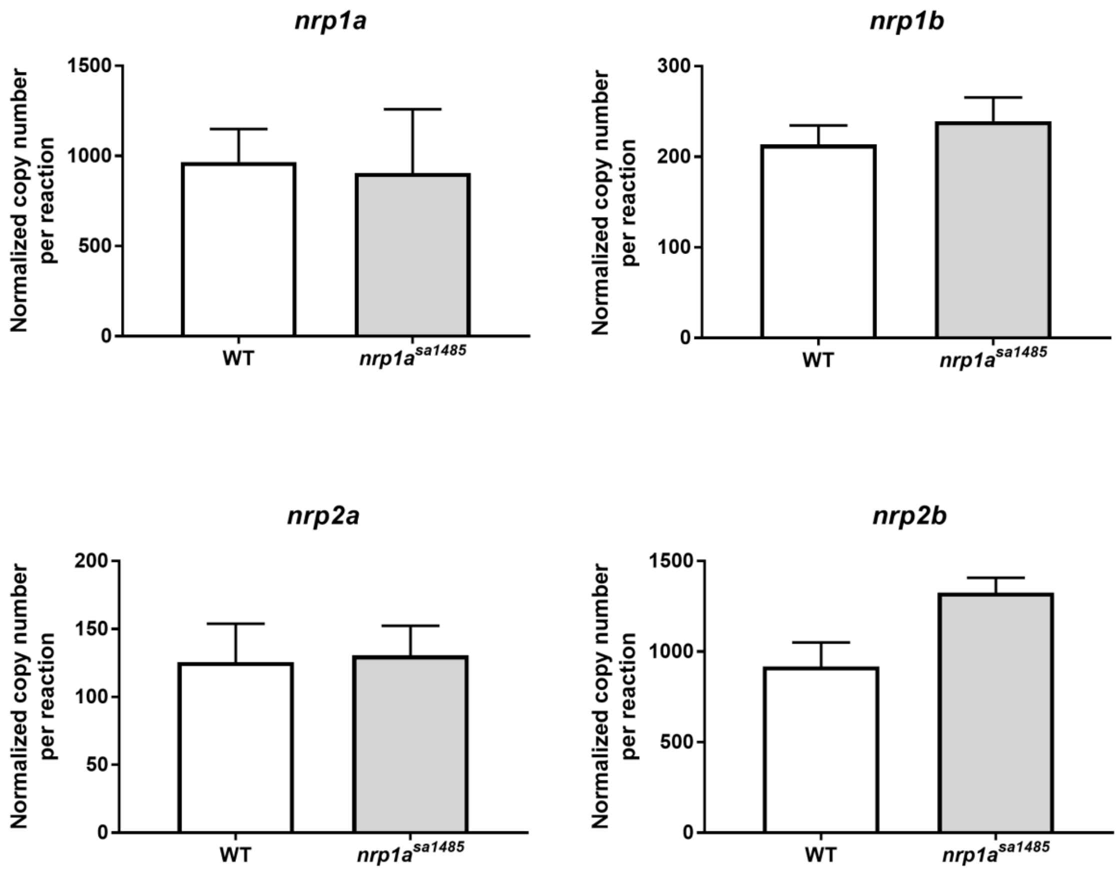

Figure S9 Gene expression of all neuropilin isoforms remains unchanged in *nrp1a*^{sa1485} fish after cryoinjury.

Absolute quantitative PCR analysis at 3 days following cryoinjury of Wild-Type or *nrp1a*^{sa1485} zebrafish ventricles. Bars represent means of normalized copy numbers per reaction ± S.E.M, (two-tailed *t*-tests of n= 6 for Wild-Type and n=4 for *nrp1a*^{sa1485} fish, each n is one heart, p>0.05).

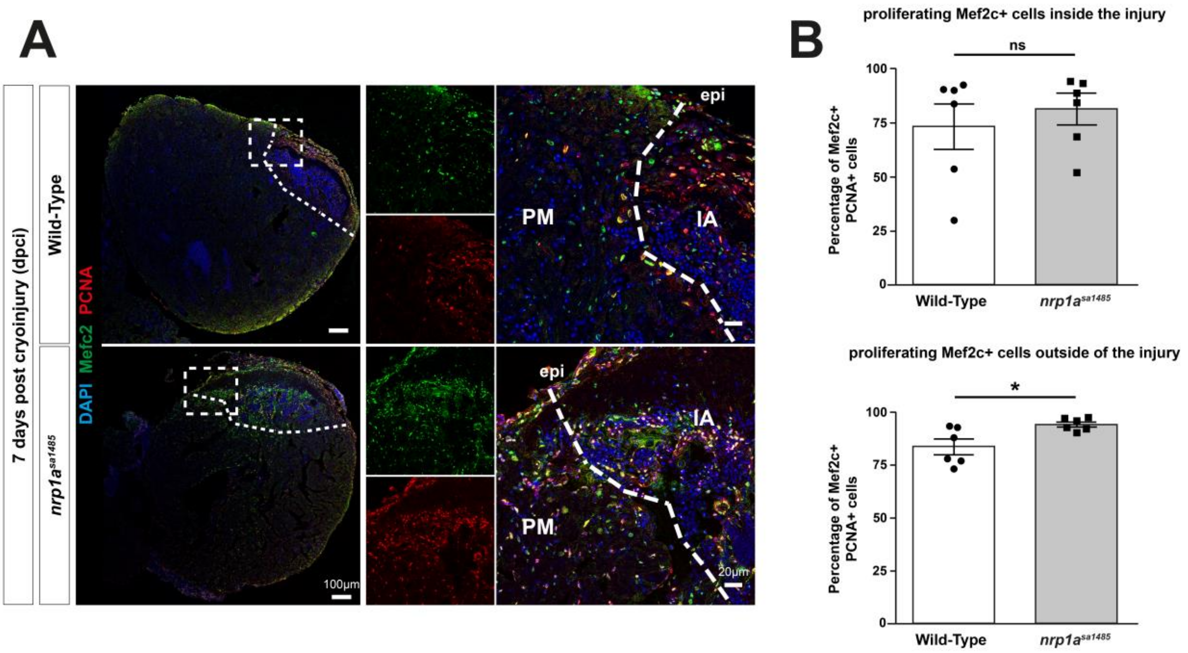

Figure S10 Cardiomyocyte proliferation of *nrp1a*^{sa1485} mutant is not affected following cryoinjury.

Sections of Wild-Type and *nrp1a*^{sa1485} mutant cryoinjured hearts (7 dpci) were examined by immunofluorescent staining for Mef2c and PCNA and counterstained with DAPI. The percentage of PCNA+ cardiomyocytes (i.e., also Mef2c-positive) inside the injury as well as at the injury periphery were quantified. Data points represent average values of individual hearts, obtained from 2-3 sections per heart, (two-tailed *t*-test of n=6, p=0.542 inside the injury, Mann-Whitney non parametric test of n=6, p=0.065 in the periphery). PM – peripheral myocardium to the injury, IA – injured area, epi – epicardium

Development • Supplementary information

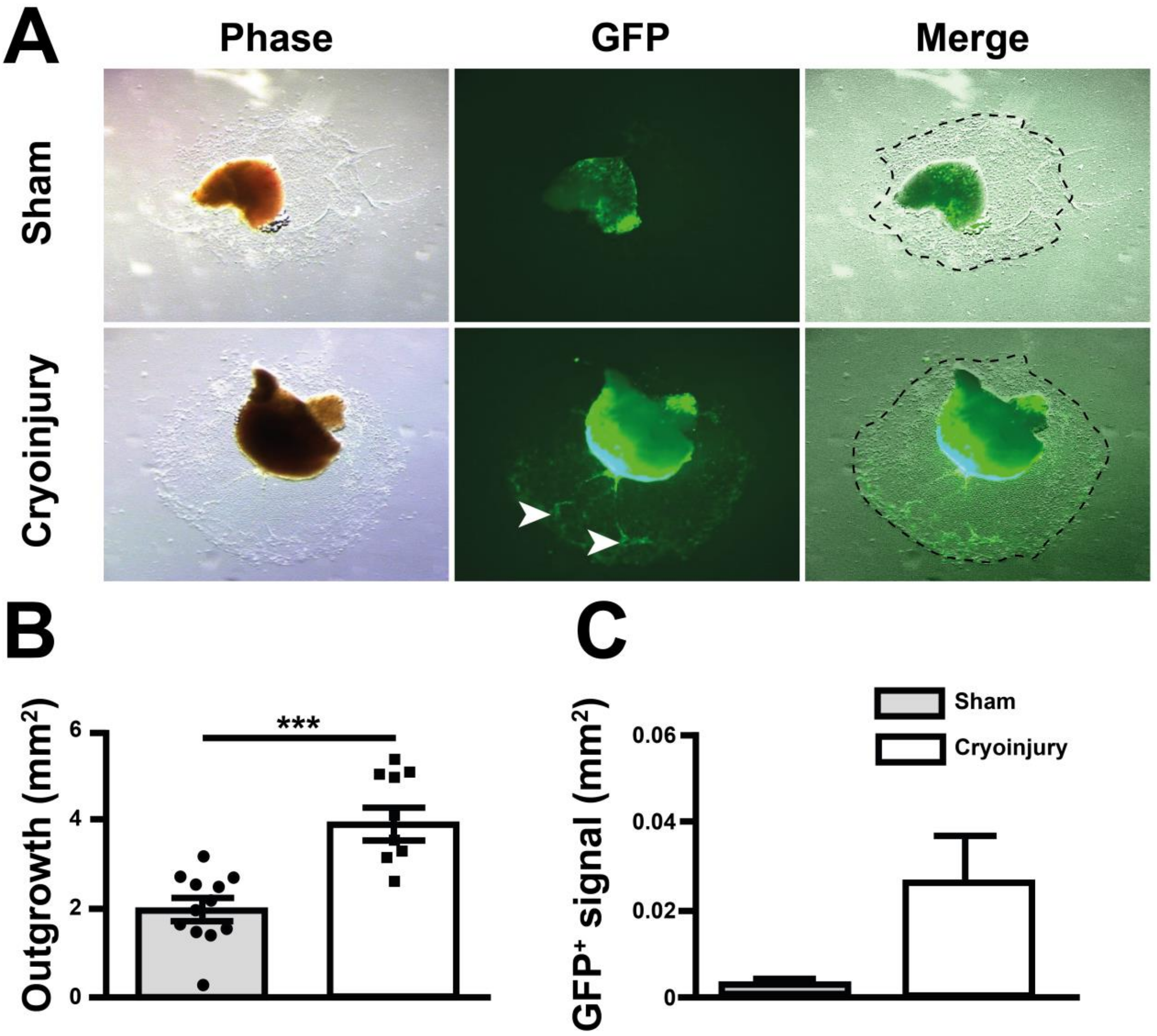

Figure S11 Cryoinjury induces epicardial activation and expansion in heart explants.

The apices of *tg(wt1b:EGFP)^{li1}* zebrafish ventricles were collected 5 days post sham surgery or cryoinjury and cultured on fibrin gels for 7 days. **(A)** Explants were imaged using a stereomicroscope under phase contrast and green-fluorescence, then overlaid to visualize GFP-positive epicardial outgrowth (white arrows), scale bar 500µm. **(B)** Epicardial outgrowths from sham (white bar) and cryoinjury (grey bar) surgeries were quantified. Results are presented as mean outgrowth in mm^2 ± S.E.M (two-tailed *t*-test of n = 12 sham and n = 10 cryoinjury, ***p=0.0003). **(C)** GFP+ signal in the epicardial outgrowth was measured for sham (white bar) and cryoinjury (grey bar) surgeries. Results are presented as mean GFP+ signal area in mm^2 ± S.E.M (two-tailed *t*-test of n = 4, p=0.0713).

Development • Supplementary information

Gene	Name	Accession Number	Primer Sequence 5’-3’
rpl13a	Ribosomal protein L 13A	NM_212784	Fwd gtctgaaacccacacgcaaat<br>Rev cgttcttttcagcctgcttagt
eef1a1a	Eukaryotic translation elongation factor 1a	NM_200009	Fwd ctcctctgggtcgttttgct<br>Rev tatgtgtctctggagttggca
gapdh	Glyceraldehyde 3-phosphate dehydrogenase	NM_001115114	Fwd ttcctgagctcaatggcaagc<br>Rev agacggactgtcagatccaca
nrp1a	Neuropilin 1a	NM_001040326 & NM_181497	Fwd ctccaacaaaccctaccaggt<br>Rev tcggtgatgtccaccatgatttc
nrp1b	Neuropilin 1b	AY493415	Fwd gaccaaaagcagatggagggaa<br>Rev catcttctgtattctctggatctttgc
nrp2a	Neuropilin 2a	NM_212965	Fwd gattctgacttcagctgggttatg<br>Rev cgatgtacaggtagtttcccaaa
nrp2b	Neuropilin 2b	NM_212966	Fwd cagcattgagcttgagcagt<br>Rev tcaggtcttcgctcagtcat
kdrl	Kinase insert domain receptor like	NM_131472	Fwd ccttgagacgcagatgaatcc<br>Rev ctgcgttatccaccctggtc
flt1	Fms-related tyrosine kinase 1	NM_001014829 & NM_001257153	Fwd aactcacagaccagtgaacaaga<br>Rev ttagccttctgtgggtatgtcca
vegfaa	Vascular endothelial growth factor Aa	NM_001190933	Fwd ccatctgtctgctgtaaaggct<br>Rev gatgatgtctaccagcagctctc

Development • Supplementary information

vegfc	Vascular endothelial growth factor c	NM_205734	Fwd tgccatgcaggagcattcaga<br>Rev gcctcctccgaccttgtttc
tgfb1a	Transforming growth factor beta 1a	NM_212965	Fwd gctggctctcatttgacgtg<br>Rev ctctgcttgtctagccctga
pdgfra	Platelet-derived growth factor receptor alpha	NM_131459	Fwd tgataatctctacacaacgctgagt<br>Rev ctcatagacatcactggacgcat
pdgfrb	Platelet-derived growth Factor receptor beta	NM_001190933	Fwd gctatcacaacaggactggttc<br>Rev ctctgtgcgaaagtctggga
pdgfab	Platelet derived growth factor alpha b	NM_001076757	Fwd tgataatctctacacaacgctgagt<br>Rev caggggtctgaggtcaaagtc
aldh1a2	retinaldehyde dehydrogenase 2	NM031850	Fwd gcgatgacctccagtgaagttg<br>Rev accttgccgctcacagaatcat

Table S1 RT-qPCR primers used for zebrafish gene expression analyses

Development • Supplementary information

Supplementary Materials and Methods:

Zebrafish husbandry, cryoinjury and sample collection

Procedures were performed in line with the Animals (Scientific Procedures) Act 1986, and husbandry was regulated by the Central University College London fish facility. Adult zebrafish between 6-18 months of age were used for the study. Wild-Type ABxTupLF (ABxTübingen-long fin) (Max-Planck, Tübingen, Germany) and *nrp1a*$^{sa1485/sa1485}$ (Zebrafish Mutation Project, Sanger Center, Cambridge, UK) fish were used for *in vitro* culture, histological samples, RNA, and protein expression analysis. Additionally, the following transgenic fish were used for histological and *in vitro* preparations: *Tg(fli1a:EGFP)*^{y1} and *tg(kdrl:mCherry)*^{s896} to identify endocardial cells, *tg(wt1b:EGFP)*^{li1} to detect activated epicardial cells. TraNac zebrafish embryos (gift from Paul Frankel, University College London) were used for whole-mount *in situ* hybridization probe validation.

The cryoinjury procedure was carried out as described in (Gonzalez-Rosa and Mercader, 2012). Briefly, fish were anaesthetized and a small incision was made to expose the ventricle. A stainless steel probe (0.75 mm diameter), cooled in liquid nitrogen, was pressed onto the ventricle apex for 5 seconds. Sham surgeries were performed as above, with the absence of probe application.

For RNA extraction, ventricles were rinsed briefly in PBS and stored at -20°C in RNAlater® stabilization reagent (Qiagen). For protein extraction, ventricles were snap frozen in liquid nitrogen and stored at -80°C until processing. For histological preparations, the entire heart (atrium, ventricle and *bulbus arteriosus*) was kept intact and placed in PBS/0.1 M KCL to arrest heart in diastole before fixing with 4% (wt/vol) paraformaldehyde overnight at 4°C. After fixation, hearts were rinsed several times in PBS, dehydrated in graded concentrations of ethanol solutions and embedded in

paraffin wax. Sections were cut in serial sections at 10 µm to slides. Sections used for Acid Fuchsin Orange G (AFOG) staining and immunohistochemistry were mounted to Superfrost® Plus slides and stored at room temperature, while sections prepared for *in situ* hybridization were mounted to Superfrost® ultra plus (both Thermo Fisher Scientific) and stored at -80°C for *in situ* hybridization procedures. For whole-mount *in situ* preparations, embryos were collected 48 hours post fertilization and fixed with 4% (wt/vol) paraformaldehyde overnight at 4°C. The following day, embryos were rinsed several times with PBS, dechorionated, and stored in 100% methanol at -20°C until processing.

RT-qPCR

Five ventricles from corresponding time points and treatments were pooled for RNA extraction and homogenized in lysis buffer (Qiagen) in 1.4mm ceramic bead-containing tubes and mechanically disrupted in a Minilys homogenizer (Peqlab). Homogenates were passed through a QIAshredder spin column (Qiagen) then total RNA was extracted using the RNeasy Mini Kit (Qiagen). The quantity and purity of RNA were evaluated using a NanoDrop 2000 spectrophotometer (Thermo Fisher Scientific) and RNA integrity was assessed with a Bioanalyzer (Agilent). 250-500 ng of total RNA was reverse transcribed using the QuantiTect® Reverse Transcription Kit (Qiagen).

All primers (see table S1) and standards were purchased from qStandard: Absolute RT-qPCR values were measured using Brilliant III Ultra-Fast QPCR Master Mix (Agilent Technologies Inc.) and the Rotor-Gene PCR thermocycler (Qiagen). Values were normalized using a normalization factor generated using GeNorm software from the following three reference genes: *gapdh*, *rpl13a* and *eef1a1a* to calculate the expression of the genes of interest.

Development • Supplementary information

Immunofluorescence

Whole heart sections were rehydrated in graded concentrations of ethanol solutions. Citrate buffer heat-induced antigen retrieval was performed (10 minutes) and samples were permeabilized in 0.5% Triton X-100 for 15 minutes followed by blocking for 1 hour at room temperature in blocking solution (PBS 0.1%Tween-20 (PBST) supplemented with 5% BSA and 10% donkey serum). Primary antibodies were diluted in blocking solution and incubated overnight at 4°C. The following antibodies were used for immunofluorescence: anti-Neuropilin 1, Abcam ab81321 (1:100) anti-WT1 6F-H2, Novus NB110-6001 (1:100); anti-GFP, Roche 11 814 460 001 (1:200); anti-Tropomyosin, sigma T2780 (1:100); anti-mCherry, Novus NBP1-96752 (1:100); anti-PCNA PC-10, Santa Cruz sc-56 (1:100); anti-ALDH2 (RALDH2), Genetex GTX101429 (1:100); anti-Mef2, biorbyt orb256682(1:100); normal rabbit IgG, alpha diagnostics 200009-1-200 (1:200); normal mouse IgG, Invitrogen 026502 (1:200). The following day, slides were washed in PBST and incubated with fluorescent secondary antibodies: anti-rabbit Alexa 488 and anti-mouse Alexa 555 (both Thermo Fisher Scientific, A31570 and A11034) for 1 hour at room temperature. Slides were washed with PBST and incubated in 1% (wt/vol) Sudan Black B in 70% ethanol for 15 minutes at room temperature to quench background fluorescence and rinsed 8 times rapidly with PBS before mounting with ProLong DAPI mounting medium (Thermo Fisher Scientific). Images were captured on the Leica TCS SPE1 confocal microscope system and processed using the publicly available ImageJ software.

AFOG staining

Deparaffinized and rehydrated sections were fixed in Bouins fixative (Thermo Fisher Scientific) for 2 hours at 60°C followed by overnight incubation at room temperature. The following day, slides were washed for 10 minutes in constant tap water stream. Nuclei were

Development • Supplementary information

stained with Weigert's iron hematoxylin (Amresco, Sigma-Aldrich), and treated for 5 minutes with 1% phosphomolybdic acid in ddH_2O (Sigma-Aldrich). Slides were then incubated in AFOG staining solution (5g Methyl Blue (Sigma), 10g Orange G (Sigma-Aldrich), 15g acid fuchsin (Acros organics) per litre of double distilled water (ddH_2O), pH=1.09) for 10 minutes and washed in ddH_2O 5 times. Slides were then rapidly dehydrated in a series of ethanol solutions of increasing concentrations to a final incubation in xylene and mounted for imaging in NanoZoomer automated slide scanner (Hamamatsu).

In situ hybridization

Digoxigenin-labelled RNA (Roche) probe templates were generated from adult zebrafish heart cDNA, the primers used were as follows (5'-3'):

nrp1a fwd TACAGTGCCGCCTACTACAC, rev CACGCTTCCGAGTACGAGTT;

nrp1b fwd CAAAACCATGACACGCCAGA rev TGCCCTCACAGTTCACGATTT;

nrp2a fwd AGACCAGCACGACACAGAAA, rev GTGAGGGGTTTGGTGTGGTC;

nrp2b fwd ACCACCATTCCTGACACTGC, rev GTGAGGGGTTTGGTGTGGTC.

Previously established probes, such as: *cmlc2*, *raldh2*, *wt1b* and *tbx18* (gift from Nadia Mercader, Universität Bern, Switzerland) were synthesized in a similar manner. Primers were used in a PCR reaction to amplify probe sequence region and amplicons cloned to pGEM®-T plasmid vectors. Each plasmid was used in two RNA polymerase reactions (either SP6 or T7) to generate the sense and the anti-sense probes.

After deparaffinization in xylene, sections were rehydrated, fixed with 4% (wt/vol) paraformaldehyde at room temperature for 10 minutes and digested with proteinase K (10µg/ml in PBS) at 37°C for 10 minutes. Sections were then post-fixed with 4% (wt/vol) paraformaldehyde for 5 minutes at room temperature, washed twice in PBS, and acetylated for 10 minutes with 0.25% acetic anhydride in 0.1 M triethanolamine (DEPC-treated), then

washed in PBS. Hybridization solution (50% deionised formamide, 10% dextran sulphate, 1X Denhardt's, 5X Saline-Sodium Citrate (SSC), 1 mg/ml yeast tRNA, 0.1% Tween 20) was placed on samples and incubated at 67°C for 2 hours in a humidifying chamber. Either anti-sense (AS) or sense (S) digoxigenin-labelled probes (0.5µg probe/ml) in hybridization solution were added to the samples overnight at 67°C in a humidifying chamber. Slides were washed at 67°C in graded salt solutions (5X SSC/50% formamide, 2X SSC/50% formamide, 2X SSC and 0.2X SSC) for 30 minutes per wash, then washed with malate buffer (100 mM maleic acid, 150 mM NaCl, pH7.5, 0.1% Tween 20) (MAB) 3 times, and blocked (MAB/ 2% Boehringer blocking reagent/10% sheep serum) at room temperature for 2 hours. Alkaline phosphatase-conjugated anti-digoxigenin antibody (Roche) was incubated on samples overnight at 4°C and, the following day, slides were washed with MAB and equilibrated in staining buffer (100 mM Tris pH9.5, 50 mM $MgCl_2$, 100 mM NaCl, 0.1% Tween 20). Gene detection by alkaline phosphatase/NBT/BCIP reaction (containing 5% polyvinyl alcohol) was carried out at 37°C in the dark until a signal was detected. Slides were fixed with 4% (wt/vol) paraformaldehyde before rapid dehydration in graded concentrations of ethanol solutions to xylene for imaging in NanoZoomer automated slide scanner (Hamamatsu).

Neuropilin probes were validated with whole-mount *in situ* hybridization of dechorionated embryos. Embryos were rehydrated in graded concentrations of methanol, permeabilized with proteinase K (10 µg/ml) at RT for 20 minutes and further fixed in 4% (wt/vol) paraformaldehyde. Hybridization buffer (50% formamide, 5X Saline-Sodium Citrate buffer (SSC), heparin (50 µg/ml), torula yeast tRNA (5 mg/ml), 0.1% Tween 20) was used to block embryos at 67°C for one hour. Probes were diluted to 0.5 µg probe/ml in the same hybridization buffer and denatured at 80°C for 3 minutes. Equilibrated embryos were then incubated in probe-containing hybridization solution at 67°C overnight. Similarly, detection was performed using alkaline phosphatase-conjugated anti-DIG antibodies (Roche).

Visualization was done using alkaline phosphatase substrate BM purple (Roche) in the dark at room temperature until a dark purple precipitant developed. Embryos were then post fixed with 4% (wt/vol) paraformaldehyde overnight at 4ºC and stored in 80% glycerol/PBS for microscopic analysis.

In vitro epicardial cell culture (heart explants)

Thrombin/fibrin reaction in Dulbecco's Modified Eagle Medium (DMEM, Thermo Fisher Scientific) was performed as described previously (Kim et al., 2012) to produce a fibrin gel matrix in either 24-well plates or Lab-Tek™II 4-chamber slides (Thermo Fisher Scientific). The apex of cryoinjured and sham-operated zebrafish hearts 5 days post-surgery were isolated and rinsed several times with HDMEM (23mM HEPES, 15mM NaCl in DMEM) to remove residual blood. One apex per well/chamber was placed firmly on set fibrin gel matrices, ensuring epicardial surface contact with the gel. Excess HDMEM was removed from heart tissue and left to adhere for 1 hour in a tissue culture incubator (28°C, 5% CO_2) before careful addition of DMEM supplemented with 0.5% fetal bovine serum (FBS), Normocin™ (invivoGen) and penicillin/streptomycin antibiotics (Sigma-Aldrich) into the wells or slide chambers. Medium was changed every 2 days and cells were cultured for 7 days before harvesting epicardial outgrowths for immunofluorescence imaging. For immunofluorescence analysis, heart tissue was discarded and cells were fixed in 4% (wt/vol) paraformaldehyde for 15 mins and permeabilized with 0.1% Triton X-100 for 10 mins before blocking in PBST (1% BSA, 10% donkey serum) at room temperature for 1 hour and incubated overnight at 4°C with primary antibody diluted in blocking solution. The antibodies used to stain epicardial cells from heart explants are the following: anti-Neuropilin 1, Abcam ab81321 (1:100); anti-GFP, Roche 11 814 460 001 (1:400); anti-ALDH2, Genetex GTX124302 (1:200); normal rabbit IgG, alpha diagnostics 200009-1-200 (1:400); normal mouse IgG, Invitrogen 026502 (1:400).

Development • Supplementary information

The following day, samples were incubated for one hour at room temperature with fluorescent-tagged secondary antibodies (anti-rabbit Alexa 488 and Alexa Fluor 555 Phalloidin, both Thermo Fisher Scientific). Slides were mounted with ProLong DAPI mounting medium (Thermo Fisher Scientific).

Immunoblotting

Lysates were obtained from zebrafish hearts by homogenizing 3 ventricles in RIPA buffer (Sigma-Aldrich) supplemented with TCEP, protease inhibitor cocktail (Roche) and phosphatase inhibitor cocktails (Sigma-Aldrich) in 1.4mm ceramic bead-containing tubes using the Minilys homogenizer (Peqlab). Lysates were supplemented with Lithium dodecyl sulfate anionic detergent (Thermo Fisher Scientific) and RIPA buffer to load equal amount of proteins. All samples were then denatured by heating for 3 minutes at 95°C before loading to gels. Proteins were separated by electrophoresis on 4–12% Bis-Tris polyacrylamide gels, and electrotransferred to PVDF membranes (all Thermo Fisher Scientific). Membranes were blocked with 5% (w/v) non-fat dried milk in PBS containing 0.1% Tween 20 (PBST), incubated with primary antibodies in PBS/blocking solution overnight at 4°C, washed five times in PBST, incubated for 1 hour with horseradish peroxidase-labelled IgG (Santa Cruz biotechnology, Inc.) at room temperature. The antibodies used for immunoblotting are the following: anti-Neuropilin 1, Abcam ab81321; anti-GAPDH V-18, Santa Cruz sc-20357; β actin clone AC-15, Sigma-Aldrich, Cat No. A5441; β catenin, Sigma-Aldrich, Cat No. C2206; all used at 1:100 dilution. Proteins were detected using the ECL Plus™ Western blotting detection system and Hyperfilm (both Amersham).

Development • Supplementary information

Recombinant adenovirus generation

All reagents used for the generation of the adenoviruses constructs were from Life Technologies™.

Rat specific NRP1 shRNA construct was generated as previously described (Pellet-Many et al., 2015) using the BLOCK-iT™ U6 RNAi Entry Vector Kit, the primers used to form the hairpins are listed below. The shRNA cassette was recombined into the pAd/BLOCK-iT™-DEST vector and virus produced as described above. The primers used to generate the construct were:

Ad.shNRP1:

5'- CACCGCAGCATCTCTGAAGATTTCACGAATGAAATCTTCAGAGATGCTG -3' and,

5'- AAAAGCAGCATCTCTGAAGATTTCATTCGTGAAATCTTCAGAGATGCTGC -3'

Viral particles were released from the HEK-293A cells by three freeze-thaw cycles and purified using the Adenopure® adenovirus purification kit (Puresyn, Inc.). Purified adenoviruses were dialyzed (Slide-A-Lyzer Dialysis kit: 10,000 MWCO, 2-12 ml capacity, extra strength (Catalogue number 66807) from Thermo Scientific and stored at −20°C until needed.

Epicardial cell NRP1 knock down

Rat Epicardial cells were a generous gift from Dr Nicola Smart and described in (Wada et al., 2003). Cells were tested prior to their use in experiment and were found to be free of mycoplasma. Cells were seeded and left to adhere for 6 to 8 hours. They were then infected with shNRP1 adenovirus constructs for 24 hours as described in Pellet-Many et al. (2015). Protein lysates were harvested and immunoblotting performed as described above.

Development • Supplementary information

GONZALEZ-ROSA, J. M. & MERCADER, N. 2012. Cryoinjury as a myocardial infarction model for the study of cardiac regeneration in the zebrafish. *Nat Protoc,* 7**,** 782-8.

KIM, J., RUBIN, N., HUANG, Y., TUAN, T. L. & LIEN, C. L. 2012. In vitro culture of epicardial cells from adult zebrafish heart on a fibrin matrix. *Nature Protocols,* 7**,** 247-255.

PELLET-MANY, C., MEHTA, V., FIELDS, L., MAHMOUD, M., LOWE, V., EVANS, I., RUIVO, J. & ZACHARY, I. 2015. Neuropilins 1 and 2 mediate neointimal hyperplasia and re-endothelialization following arterial injury. *Cardiovasc Res,* 108**,** 288-98.

WADA, A. M., SMITH, T. K., OSLER, M. E., REESE, D. E. & BADER, D. M. 2003. Epicardial/Mesothelial cell line retains vasculogenic potential of embryonic epicardium. *Circ Res,* 92**,** 525-31.
